# CircHYBID regulates hyaluronan metabolism in chondrocytes via hsa-miR-29b-3p/TGF-β1 axis

**DOI:** 10.1186/s10020-021-00319-x

**Published:** 2021-05-31

**Authors:** Hong-Xing Liao, Zhi-Hui Zhang, Hui-Lin Chen, Ying-Mei Huang, Zhan-Liang Liu, Jian Huang

**Affiliations:** Orthopedic Center, Meizhou People’s Hospital, Huangtang Road No.63, Meizhou, 514000 Guangdong People’s Republic of China

**Keywords:** CircHYBID, hsa-miR-29b-3p, TGF-β1, Hyaluronan, Osteoarthritis

## Abstract

**Background:**

Hyaluronan (HA) metabolism by chondrocytes is important for cartilage development and homeostasis. However, information about the function of circular RNAs (circRNAs) in HA metabolism is limited. We therefore profiled the role of the novel HA-related circRNA circHYBID in the progression of osteoarthritis (OA).

**Methods:**

CircHYBID function in HA metabolism in chondrocytes was investigated using gain-of-function experiments, and circHYBID mechanism was confirmed via bioinformatics analysis and luciferase assays. The expression of circHYBID–hsa-miR-29b-3p–transforming growth factor (TGF)-β1 axis was examined by quantitative real-time polymerase chain reaction (qRT-PCR) and Western blotting. CircHYBID, TGF-β1, and HA levels in cartilage samples were evaluated using qRT-PCR and pathological examination. Enzyme-linked immunosorbent assay was used to assess HA accumulation in chondrocyte supernatant.

**Results:**

CircHYBID expression was significantly downregulated in damaged cartilage samples compared with that in the corresponding intact cartilage samples. CircHYBID expression was positively correlated with alcian blue score. Interleukin-1β stimulation in chondrocytes downregulated circHYBID expression and decreased HA accumulation. Gain-of-function experiments revealed that circHYBID overexpression in chondrocytes increased HA accumulation by regulating HA synthase 2 and HYBID expression. Further mechanism analysis showed that circHYBID upregulated TGF-β1 expression by sponging hsa-miR-29b-3p.

**Conclusions:**

Our results describe a novel HA-related circRNA that could promote HA synthesis and accumulation. The circHYBID–hsa-miR-29b-3p–TGF-β1 axis may play a powerful regulatory role in HA metabolism and OA progression. Thus, these findings will provide new perspectives for studies on OA pathogenesis, and circHYBID may serve as a potential target for OA therapy.

**Supplementary Information:**

The online version contains supplementary material available at 10.1186/s10020-021-00319-x.

## Background

Osteoarthritis (OA) is the most common degenerative joint disease, and its socioeconomic impact is increasing because of population aging and obesity’s rising prevalence (Lepage et al. 2019). Moreover, OA is a debilitating disease that causes functional disability in the elderly (Wallace et al. 2017). OA-associated inflammatory response is mediated by multiple factors, such as age, body weight, metalloproteinases, cytokines, signaling pathways, metabolic disorders, and noncoding RNAs (Lespasio et al. 2017; Choi et al. 2019). At present, early interventions for clinical OA mainly comprise anti-inflammatory analgesia and cartilage-protecting treatment; however, there are no effective drugs to reverse OA progression (Ghouri and Conaghan 2020).

The most important OA characteristics include degradation of the cartilage extracellular matrix (ECM) and secondary osteogenesis (Lepage et al. 2019). Cartilage ECM is a highly specialized and dynamic three-dimensional scaffold, in which chondrocytes reside. ECM comprises a variety of fibrillar components, such as collagens, fibronectin, and elastin, as well as nonfibrillar molecules, such as proteoglycans, hyaluronan (HA), and glycoproteins including matricellular proteins (Theocharis et al. 2019). Homeostasis of ECM components, including HA, is tightly controlled by synthesis and degradation in cartilage tissue, and these processes are altered under pathological conditions. The initial pathological modification of articular cartilage in patients with OA is the depletion of the HA-aggrecan (a major cartilage proteoglycan) network structure, which is followed by the degradation of collagen fibrils (Nagase and Kashiwagi 2003). HA is a nonsulfated glycosaminoglycan comprising disaccharide units of *N*-acetylglucosamine and glucuronic acid (Avenoso et al. 2020). HA metabolism plays an important role in maintaining tissue homeostasis and regulating cell behavior (Yoshino et al. 2018; Avenoso et al. 2018). However, the regulatory mechanism of HA metabolism in chondrocytes remains unclear. Elucidation of the mechanism regulating HA metabolism in chondrocytes is expected to provide a novel theoretical basis for the pathogenesis of OA.

Circular RNAs (circRNAs) are endogenous noncoding RNAs that play central regulatory roles by functioning as miRNA sponges or competing endogenous RNAs (ceRNAs) that naturally sequester and competitively inhibit miRNA activity (Shen et al. 2019). Studies have shown that multiple circRNAs that are differentially expressed in cartilage tissue play central regulatory roles in ECM degradation, inflammatory response, apoptosis, and intracellular signaling pathways (Zhou et al. 2018, 2019; Li et al. 2017). Another study revealed that the downregulation of circ_001653/miR-486-3p/cell migration-inducing hyaluronan binding protein [CEMIP] axis promoted the proliferation of NPCs and ECM synthesis of nucleus pulposus cells in intervertebral disc degeneration (Cui and Zhang 2020). CEMIP, also known as HYBID, is closely associated with cellular HA metabolism (Yoshida and Okada 2019). However, there is no clear evidence to show whether circRNA can regulate HA metabolism in cartilage tissues.

Therefore, this study investigated the function of circRNAs in HA metabolism. The findings revealed a small number of up- and downregulated circRNAs from different regions of cartilage tissue. Of interest, we specifically propose a novel circRNA, named circHYBID (has_circ_00003893), which is related to HA metabolism in chondrocytes, and systematically explored its role in OA pathogenesis. We present the following article in accordance with the Materials Design Analysis Reporting checklist.

## Methods

### Patients and specimens

All cartilage with primary damage and the corresponding normal cartilage were isolated from patients who underwent total knee replacement. Within 24 h of cartilage isolation, the articular cartilage of femoral heads was separated using a knife blade. Chondrocytes were separated using a primitive enzyme digestion method for primary culture and subculture, and the remaining cartilage tissue was frozen in liquid nitrogen for subsequent histological examination. Informed consent was obtained from all patients who underwent total knee replacement. This study was approved by the Human Ethics Committee of Meizhou People’s Hospital.

### Histological examination

The isolated cartilage samples were fixed in 4% paraformaldehyde at 4 °C for 2 days and then decalcified in 15% ethylenediaminetetraacetic acid for 2 weeks. Cartilage specimens were dehydrated with ethanol and xylene, embedded in paraffin, and cut into 5 μm sections. The prepared sections were stained with hematoxylin & eosin (HE), Safranin O, and toluidine blue according to routine protocols. Cartilage specimens were graded according to the Mankin score. Scores < 4 and > 6 indicated that the cartilage samples were intact and damaged, respectively (Liu et al. 2017).

#### Alcian blue staining

Cartilage specimens sections were put into 1% alcian blue (Sigma, St. Louis, MO, USA) for 50 min. In which, alcian blue was diluted in 3% acetic acid (Beyotime, Shanghai, China). Then sections were washed in water. Alcian blue score was evaluated following with previous study base on the grading system from zero to three: 0, negative; 1, weakly positive; 2, positive; 3, strongly positive (Gutiérrez et al. 2012). All evaluations were performed in a double-blind manner by two expert (Liao and Zhang).

### Immunohistochemistry (IHC)

IHC was performed according to a previous study (Ariffin et al. 2020). The primary antibody (Anti-TGF beta 1, #ab92486) was provided by Abcam (Cambridge, MA, USA), and second antibody was provided by Cusabio (Wuhan, China). To evaluate the expression of transforming growth factor (TGF)-β1, a semiquantitative scoring index reflecting the intensity of staining and percentage of positive cells was used (He et al. 2012). The staining index (ranging from 0 to 12) was determined as the intensity of HA or TGF-β1 staining (0, negative; 1, weakly positive; 2, positive; 3, strongly positive) multiplied by the proportion of immunopositive cells (0, 0%; 1, < 10%; 2, 10–49.9%; 3, 50–74.9%; 4, ≥ 75%). All histological evaluations were performed in a double-blind manner by two expert pathologists (Liao and Zhang).

### RNA sequencing and differential expression analysis

After RNA isolation by TRIzol (Thermo Fisher Scientific, Waltham, MA, USA), total RNA in both sample types was quantified using Qubit RNA Assay Kit (Life Technologies, Waltham, MA, USA), and the quality of RNA was assessed using Agilent 2100 Bioanalyzer and RNA6000 Nano Kit (Agilent Technologies, Santa Clara, CA, USA). The sequencing library was prepared according to the manufacturer’s instructions for NEBNext Ultra™ RNA Library prep kit (NEB, Beijing, China) for the Illumina platform. Next, we analyzed differentially expressed genes. OA and normal cartilage were selected using log2 ratio ≥ 1 and the false discovery rate of multiple hypothesis testing (p < 0.05).

### Bioinformatics analysis

CircHYBID was selected to construct the circRNA–miRNA–mRNA network. The interactions of circRNAs and mRNAs with miRNAs were predicted using prediction software based on TargetScan (http://www.targetscan.org/vert_72/) (Liu et al. 2017).

### Primary culture of chondrocytes

Donor chondrocytes were isolated as previously described (Oseni et al. 2013). Chondrocytes (5 × 10^5^/well) were obtained at passage 2 and cultured in ProNectin F-coated Bioflex six-well culture plates (Flexcell International) to 80% confluence (Liu et al. 2017). The chondrocytes were treated with 10 ng/ml of interleukin (IL)-1β in the culture medium for 12 h as per a previous study (Wu et al. 2020). Untreated chondrocytes were used as controls.

### Plasmid construction and transfection

CircHYBID and TGF-β1 overexpression plasmids were purchased from RiboBio (Guangzhou, China) and transfected into cells for overexpressing circHYBID and TGF-β1, and the vector LV003 (Forevergen, Guangzhou, China) was used as a negative control (OV-NC). The wild-type (WT) or mutated type (MUT) circHYBID and TGF-β1 sequence was cloned into pmir-GLO (Promega, China), which was performed by RiboBio (Guangzhou, China); miR-29b-3p mimics and mimic-negative control (mi-NC) were also synthesized by RiboBio for subsequent experiments. All the above plasmids were verified by direct sequencing. Lipofectamine 2000 (Invitrogen, Carlsbad, CA, USA) was utilized for all transfections as per the manufacturer’s instructions. Cells with the highest transfection efficiency were obtained after 48 h of culture for subsequent experiments.

### Quantitative real-time polymerase chain reaction (qRT-PCR)

Total RNA was isolated from cartilage tissues or primary chondrocytes using TRIzol reagent. gDNA was extracted from tissues or cultured cells using PureLink™ Genomic DNA Mini Kit protocol (Thermo Fisher Scientific). RNA was reverse-transcribed using HiScript II Q RT SuperMixfor Q-PCR (+genomic DNA [gDNA] wiper) (Vazyme, Nanjing, China). AmpliTaq DNA Polymerase (Life Technologies) was used for PCR. Then, the complementary DNA (cDNA) and gDNA PCR products were observed using 2% agarose gel electrophoresis. AceQqPCR SYBR Green Master Mix (Vazyme) was used for qPCR. For circRNA and mRNA expressions, GAPDH was used as the internal control. For miRNA expression, U6 was used as the internal control. All primer sequences are listed in Additional file 1: Table 1. The relative expression levels were determined using the 2^−ΔΔCt^ method.

### Dual-luciferase assay

The WT/MUT plasmids of circHYBID or TGF-β1 were co-transfected into cells with miR-29b-3p mimics, respectively, and changes in the luciferase activity were analyzed using the Dual Glo Luciferase Assay System (Promega) as per the manufacturer’s instructions. Each experiment was performed in triplicate.

### Enzyme-linked immunosorbent assay (ELISA)

HA, IL-1β, IL-6, and TNF-α concentrations in the supernatant of chondrocytes were determined using ELISA kits (R&D Systems, MN, USA) according to the manufacturer’s instructions.

### Western blotting (WB)

The cells were harvested and lysed using RIPA buffer (Beyotime). Proteins were quantified using a Bradford kit (Pierce, Rockford, IL, USA) and separated on a 10% sodium dodecyl sulfate–polyacrylamide gradient gel. Proteins were then transferred to polyvinylidene fluoride membranes (Millipore, MA, USA), and the membranes were incubated in 5% bovine serum albumin for 1 h. To detect protein expression, membranes were incubated with antibodies, including anti-HAS2 (ab131364, Abcam, USA, diluted 1:2000, host species: Rabbit), anti-HAS1 (ab198846, Abcam, diluted 1:2000, host species: Rabbit), anti-HAS3 (ab170872, Abcam, diluted 1:2000, host species: Rabbit), anti-HYBID (ab98947, Abcam, diluted 1:2000, host species: Rabbit), anti-IL-1β (ab216995, Abcam, diluted 1:2000, host species: Rabbit), anti-IL-6 (ab233706, Abcam, diluted 1:2000, host species: Rabbit), and anti-TGF-β1 (3709, CST, USA, diluted 1:1000, host species: Rabbit) at 4 °C overnight. The membranes were subsequently incubated with secondary HRP-conjugated Goat Anti-Rabbit IgG H&L antibody (ab6721, Abcam, diluted 1:2000) at ambient temperature for 1 h. The HRP signal was detected using a chemiluminescence reagent (Millipore, CA, USA). Protein expression was quantified using ImageJ software (National Institutes of Health, Bethesda, MD, USA). Anti-GADPH (ab9485, Abcam, diluted 1:5000, host species: Rabbit) was used as the internal control.

### Statistical analysis

Statistically significant differences between groups were calculated using analysis of variance or the nonparametric Wilcoxon signed-rank test. The results from two groups were compared using a two-tailed paired Student’s *t*-test. The strength of the association between continuous variables was tested using Spearman’s correlation. The results are reported as means ± standard deviation. p < 0.05 was considered statistically significant. All experiments were performed and analyzed in triplicate. Data analysis was performed using SPSS 25.0 (Abbott Laboratories, Chicago, IL, USA).

## Results

### Proteoglycan of ECM and HA accumulation was significantly decreased in damaged cartilage

**Fig. 1:**
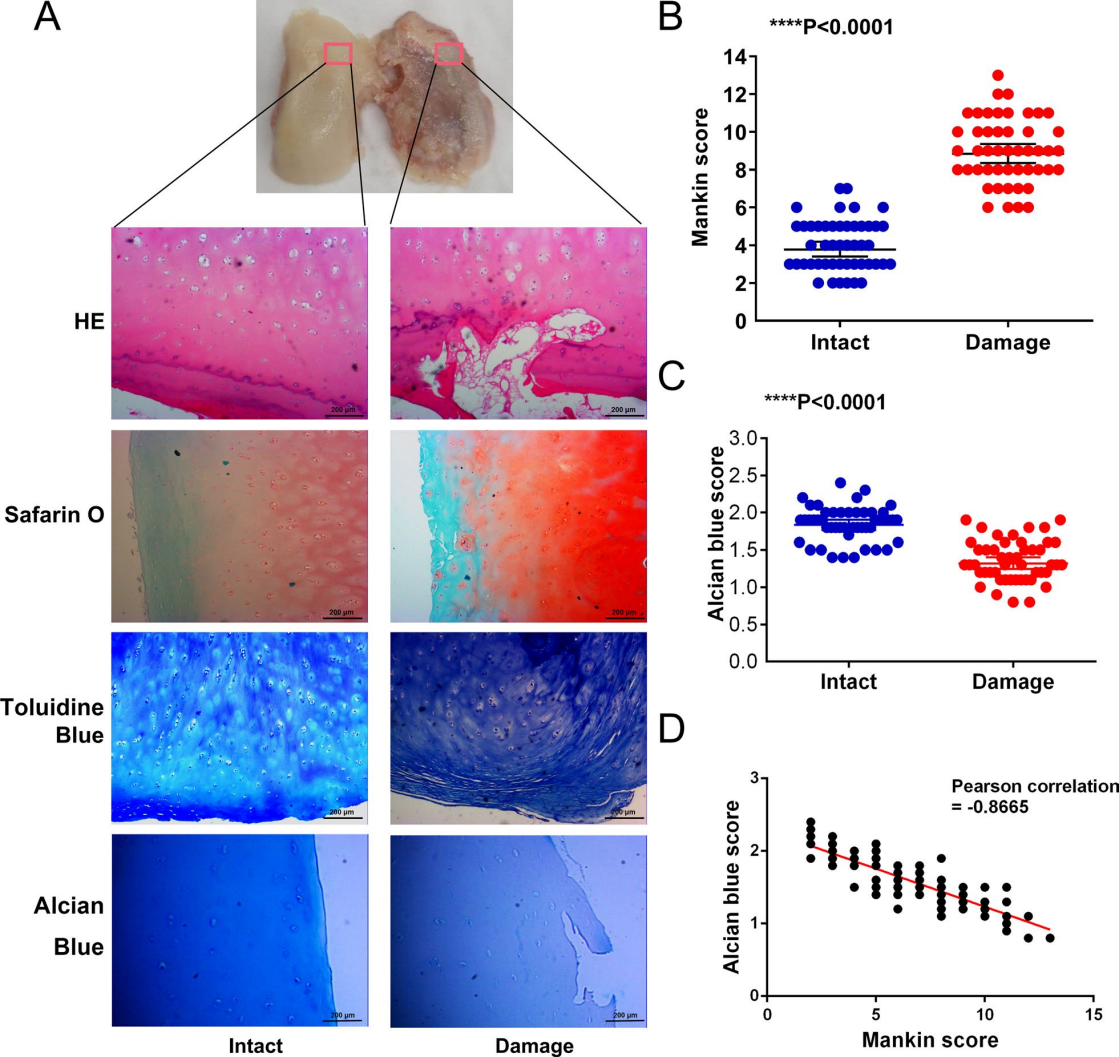
Proteoglycan of ECM and Hyaluronan (HA) accumulation was decreased in damaged cartilage. **A** Pathological examination and alcian blue staining of cartilage from intact and damaged areas (magnification × 100, scale bar 200 µm). The histological change was assessed using the Mankin score. **B** The Mankin score was significantly higher for damaged cartilage than for intact cartilage (Wilcoxon’s test, p < 0.0001). **C** The alcian blue staining score was significantly lower for damaged cartilage than for intact cartilage (Wilcoxon’s test, p < 0.0001). **D** Correlation scatter plot of the alcian blue score and the Mankin score (Pearson’s correlation, r =  − 0.8665)

To evaluated HA expression in damaged cartilage, 48 pairs of cartilage samples from intact and damages areas were histologically assessed, including HE, Safranin O, and toluidine blue staining (Fig. 1A), and graded using a modified Mankin score (Additional file 2: Table 2). The mean score for intact cartilage was 4 compared with 9 for damaged cartilage (p < 0.0001; Fig. 1B and Additional file 3: Table 3). Regarding alcian blue, proteoglycan of ECM including HA was stained as dark blue in intact cartilage, whereas the proteoglycan was stained as light blue in damaged cartilage (Fig. 1A), The median staining score of intact cartilage was 1.85 compared with 1.35 for damaged cartilage (p < 0.0001; Fig. 1C and Additional file 3: Table 3). Pearson’s analyses performed using Mankin score and alcian blue score revealed a negative correlation between the Mankin score and alcian blue score in OA cartilage tissues (Fig. 1D). The above results indicated that proteoglycan of ECM accumulation was significantly decreased in damaged cartilage and is negatively correlated with the Mankin score. These results also indirectly suggested that HA, an important component of proteoglycan, was significantly decreased in damaged cartilage.

### CircRNA expression profiles in intact and damaged cartilage

**Fig. 2:**
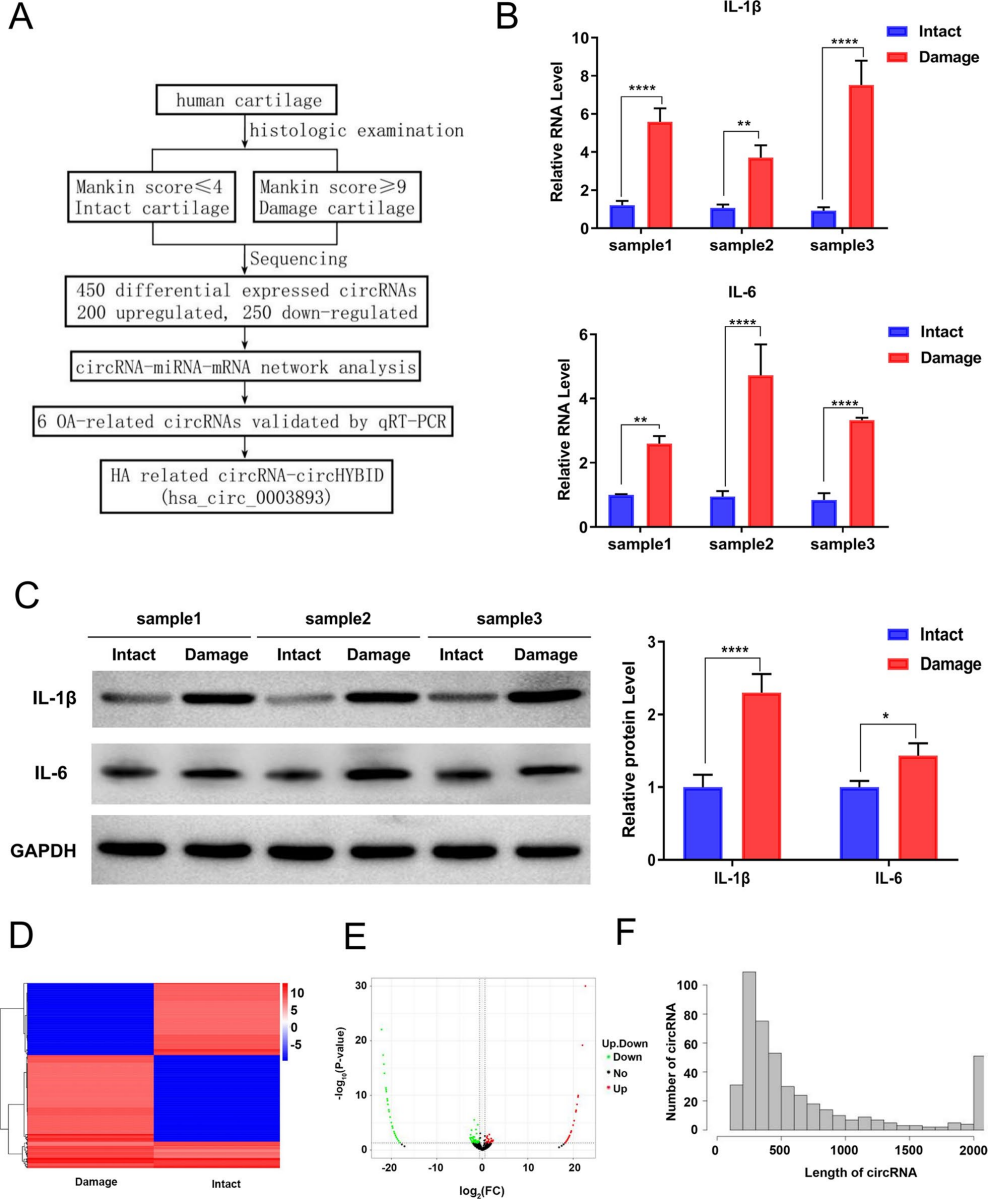
Differential expression of circular RNAs (circRNAs) in cartilage. **A** Screening flow of circRNAs. **B** Quantitative real-time polymerase chain reaction was used to assess interleukin (IL)-1β and IL-6 expression in three selected pairs of intact and damaged cartilage samples. **C** Western blotting was used to assess IL-1β and IL-6 expression in three selected pairs of intact and damaged cartilage samples. **D** Hierarchical clustering analysis of differentially expressed circRNAs between damaged and intact cartilage (> onefold difference in expression; p < 0.05). **E** The volcano map of circRNA expression (x-axis, log2 ratio of circRNA expression levels; y-axis, false discovery rate of circRNA [− log10 conversion]). **F** Frequency distribution of circRNA length (x-axis, range of circRNA length; y-axis: frequency of circRNAs of specific lengths)

Based on the histological examination, three pairs of cartilage samples were collected for further analysis (Fig. 2A). Among these selected samples, the Mankin score of intact cartilage was ≤ 4 compared with ≥ 9 for damaged cartilage (Additional file 4: Table 4). These selected samples were then used for further detection of inflammatory factors via qRT-PCR and WB analysis. The results showed that IL-1β and IL-6 expression was upregulated in damaged cartilage (Fig. 2B and C), which was further confirmed that the samples were intact cartilage and damaged cartilage, respectively.

Sequencing was subsequently performed to characterize the expression profiles of circRNAs in cartilage. We identified 450 differentially expressed circRNAs between paired intact and damaged cartilage (Additional file 5: Table 5). Then, differentially expressed circRNAs between intact and damaged cartilages were subjected to hierarchical clustering analysis and volcano plot (log2 ratio ≥ 1 and the false discovery rate of multiple hypothesis testing p < 0.05). Among these differentially expressed circRNAs, 200 were upregulated in damaged tissue samples, whereas 250 were downregulated (Fig. 2D and E). The distribution frequency of these differentially expressed circRNAs showed that most are generally 200–500 bp in length (Fig. 2F). The above results indicated that there was a difference in the expression of circRNA between intact and damaged cartilages.

### CircHYBID was predicted to be related to HA metabolism

**Fig. 3:**
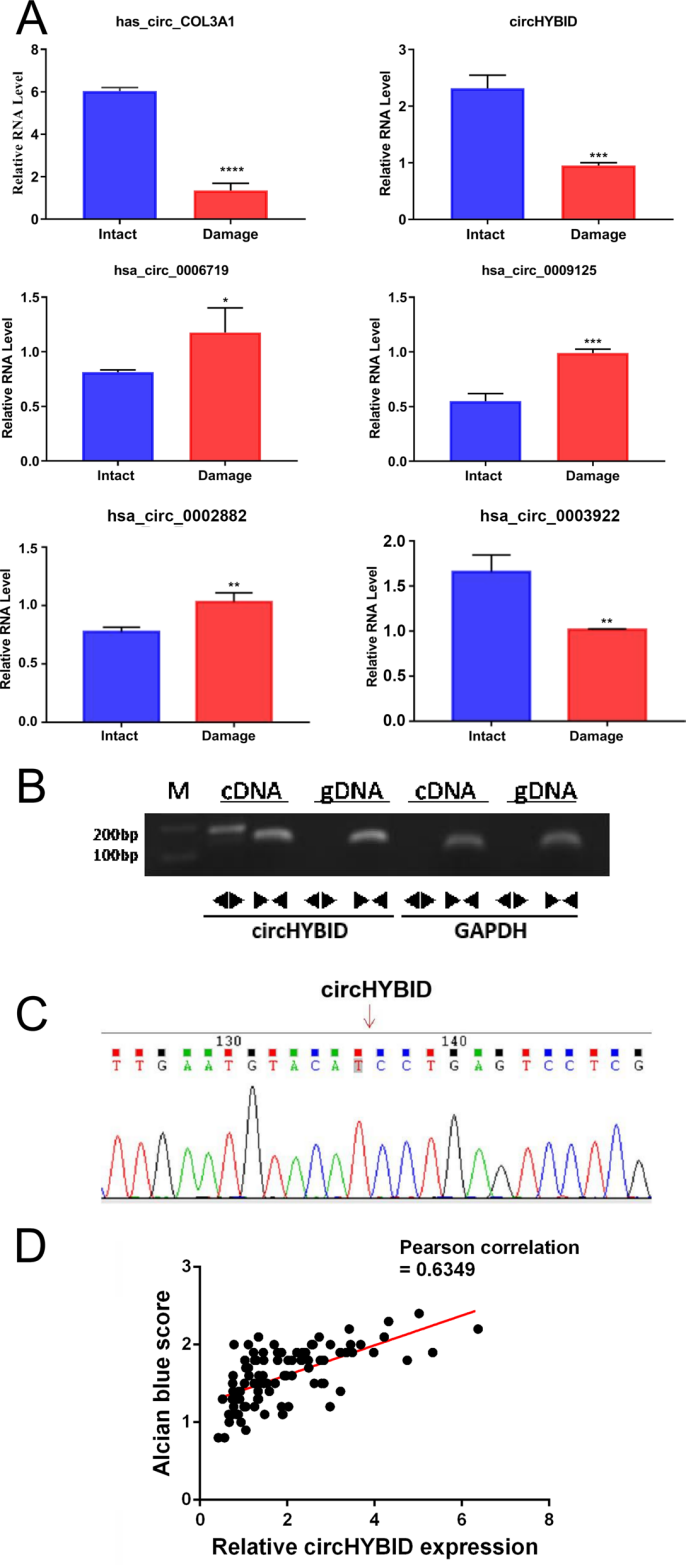
Differentially expressed circular RNAs (circRNAs) related to hyaluronan (HA) metabolism and characterization of circHYBID. **A** The expression trends of has-circ-COL3A1, circHYBID, hsa_circ_0006719, hsa_circ_0009125, hsa_circ_0002882, and hsa_circ_0003922 were confirmed by quantitative real-time polymerase chain reaction (PCR). **B** PCR analysis of circHYBID and its linear isoform HA-binding protein involved in hyaluronan depolymerization (HYBID) using complementary and genomic DNA. GAPDH was used as the linear RNA control. **C** The back-splice junction of circHYBID was identified by Sanger sequencing. **D** Correlation scatter plot of the relative expression of circHYBID and the alcian blue score (Pearson’s correlation, r = 0.6349). *p < 0.05; **p < 0.01; ***p < 0.001; ****p < 0.0001

To investigate whether circRNA can regulate HA metabolism in cartilage tissues, we first analyzed the host genes of the differentially expressed circRNAs involved in the pathogenesis of OA. The results showed that there were six circRNAs involved, which were then verified via qRT-PCR. The following circRNAs were identified: hsa_circ_0057390 (hsa_circCOL3A1, chr2:189859772|189861222), hsa_circ_0003893 (hsa_circHYBID (KIAA1199), chr15:81229015|81230320), hsa_circ_0006719 (chr16:31102096|31102663), hsa_circ_0009125 (hsa_circHABP4, chr9:99220661|99220823), hsa_circ_0002882 (hsa_circAXL, chr19:41754419|41754725), and hsa_circ_0003922 (hsa_circSP100, chr2:231307652|231314970). Among these, hsa_circCOL3A1, hsa_circHYBID, and hsa_circ_0003922 were downregulated, whereas hsa_circ_0006719, hsa_circ_0009125, and hsa_circ_0002882 were upregulated in damage cartilage compared with intact cartilage (Fig. 3A). These gene expression trends were consistent with the sequencing results (Fig. 3A and Additional file 5: Table 5). Among these circRNAs, we noted a special cartilage-associated circRNA (hsa_circHYBID (also named as hsa_KIAA1199), circBase_ID: hsa_circ_0003893 in circBase (http://circbase.org), which is a 398 bp length circRNA and is located at chr15:81229014–81230320. The host gene KIAA1199 of circHYBID encodes HA-binding protein involved in HA depolymerization (HYBID). Because HYBID was previously identified as an important enzyme that is involved in HA degradation (Nagaoka et al. 2015), we assumed that circHYBID might also play an important role in HA metabolism and OA pathogenesis. Therefore, circHYBID was chosen for further investigation.

To characterize circHYBID, divergent and convergent primers were designed to amplify the circular and linear transcripts, respectively, in both cDNA and gDNA. PCR results revealed that the circular form was amplified using the divergent primers only from cDNA, whereas convergent primers amplified both cDNA and gDNA (Fig. 3B). Furthermore, the reverse splicing of circHYBID was successfully confirmed using Sanger sequencing for the products of circHYBID divergent primers (Fig. 3C). Pearson’s analysis revealed a positive correlation between the circHYBID expression and alcian blue score (Fig. 3D). These results indicated that circRNA might regulate HA metabolism in cartilage tissues.

### CircHYBID expression and HA accumulation were downregulated after IL-1β treatment in primary chondrocytes

**Fig. 4:**
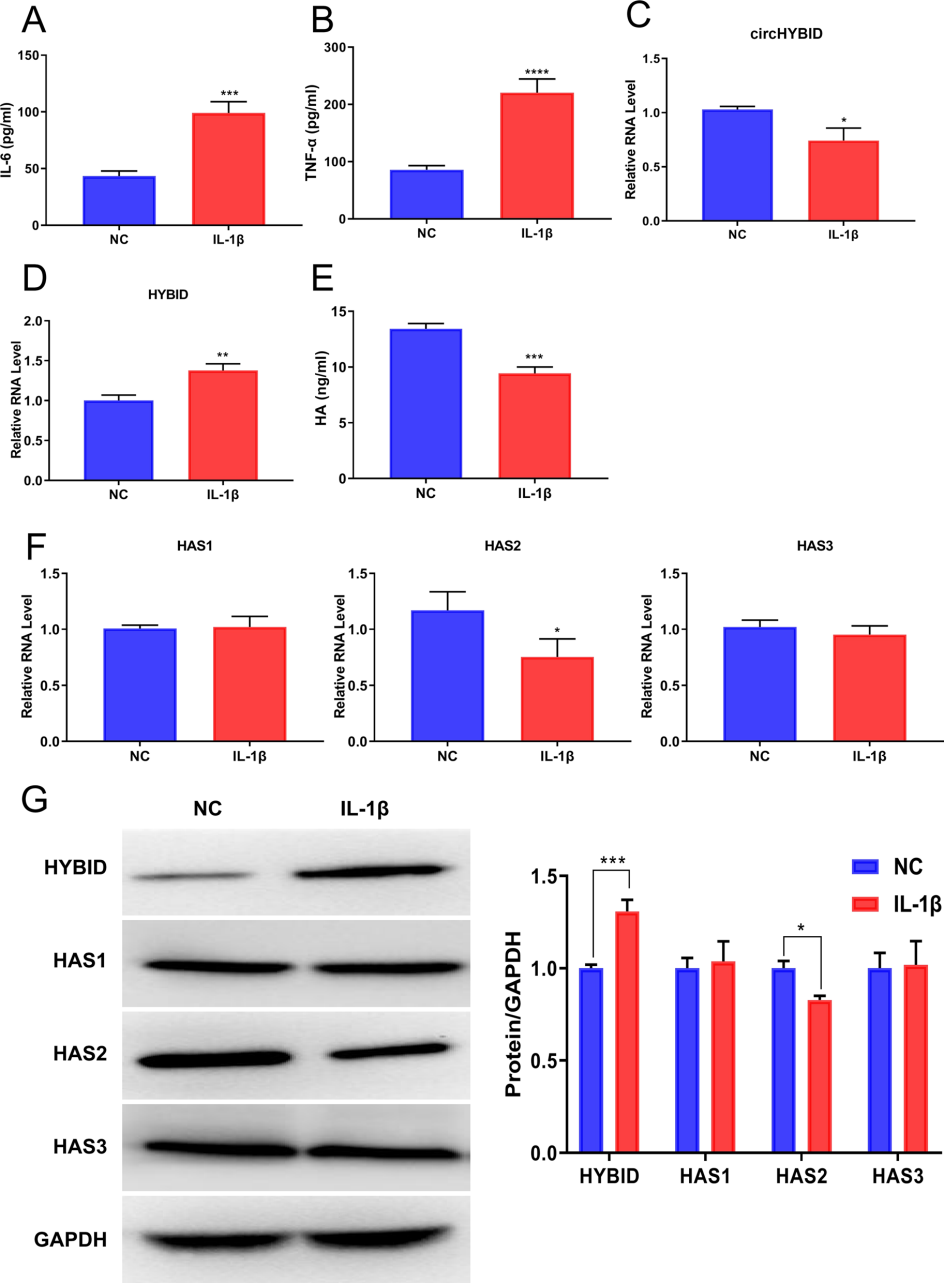
CircHYBID expression and hyaluronan (HA) accumulation were decreased by interleukin (IL)-1β treatment. IL-6 (**A**), TNF-α (**B**), and HA (**E**) levels in cell culture supernatant were measured using enzyme-linked immunosorbent assay (ELISA). The mRNA expression of circHYBID (hsa_circ_0003893) (**C**), HA-binding protein involved in hyaluronan depolymerization (HYBID) (**D**), HA synthase 1 (HAS1), HA synthase 2 (HAS2), and HA synthase 3(HAS3) (**F**) were analyzed by quantitative real-time polymerase chain reaction in chondrocytes in the presence or absence of IL-1β treatment. The protein expression of HAS1, HAS2, HAS3, and HYBID (**G**) was analyzed by Western blotting in chondrocytes in the presence or absence of IL-1β treatment. GAPDH was used as the internal control. *p < 0.05; **p < 0.01; ***p < 0.001; ****p < 0.0001

To further validate the role of circHYBID in OA, primary chondrocytes were successfully isolated (NC group) and stimulated by IL-1β to establish an OA model in vitro (IL-1β group) as per a previous study (Liu et al. 2016). ELISA results showed that inflammatory factors IL-6 and TNF-α’s expressions were upregulated after IL-1β stimulation, confirming that the OA model in vitro was successfully established (Fig. 4A and B). Then, circHYBID and host gene HYBID expression were detected in the in vitro OA model via qRT-PCR, and the results showed that circHYBID was downregulated after IL-1β stimulation in chondrocytes, which was consistent with the sequencing results of cartilage tissues (Fig. 4C); however, host gene HYBID was upregulated in the IL-1β group compared with the NC group (Fig. 4D and G). Moreover, HA in the supernatant was decreased after IL-1β treatment using ELISA, which was consistent with the previous IHC results of HA (Fig. 4E). Furthermore, we investigated HA synthase 1 (HAS1), HA synthase 2 (HAS2), and HA synthase 3 (HAS3) expression via qRT-PCR and WB analysis, which are involved in HA metabolism (Tammi et al. 2011). HAS2 expression was downregulated in chondrocytes treated with IL-1β (Fig. 4F and G). However, there was no significant difference in HAS1 and HAS3 expression under IL-1β stimulation (Fig. 4F and G). These results suggested that circHYBID and HA were decreased in the in vitro OA model, in which HYBID and HAS2 expression probably play an important regulatory role.

### CircHYBID overexpression increases HA accumulation after IL-1β treatment in primary chondrocytes by regulating HYBID and HAS2 expression

**Fig. 5:**
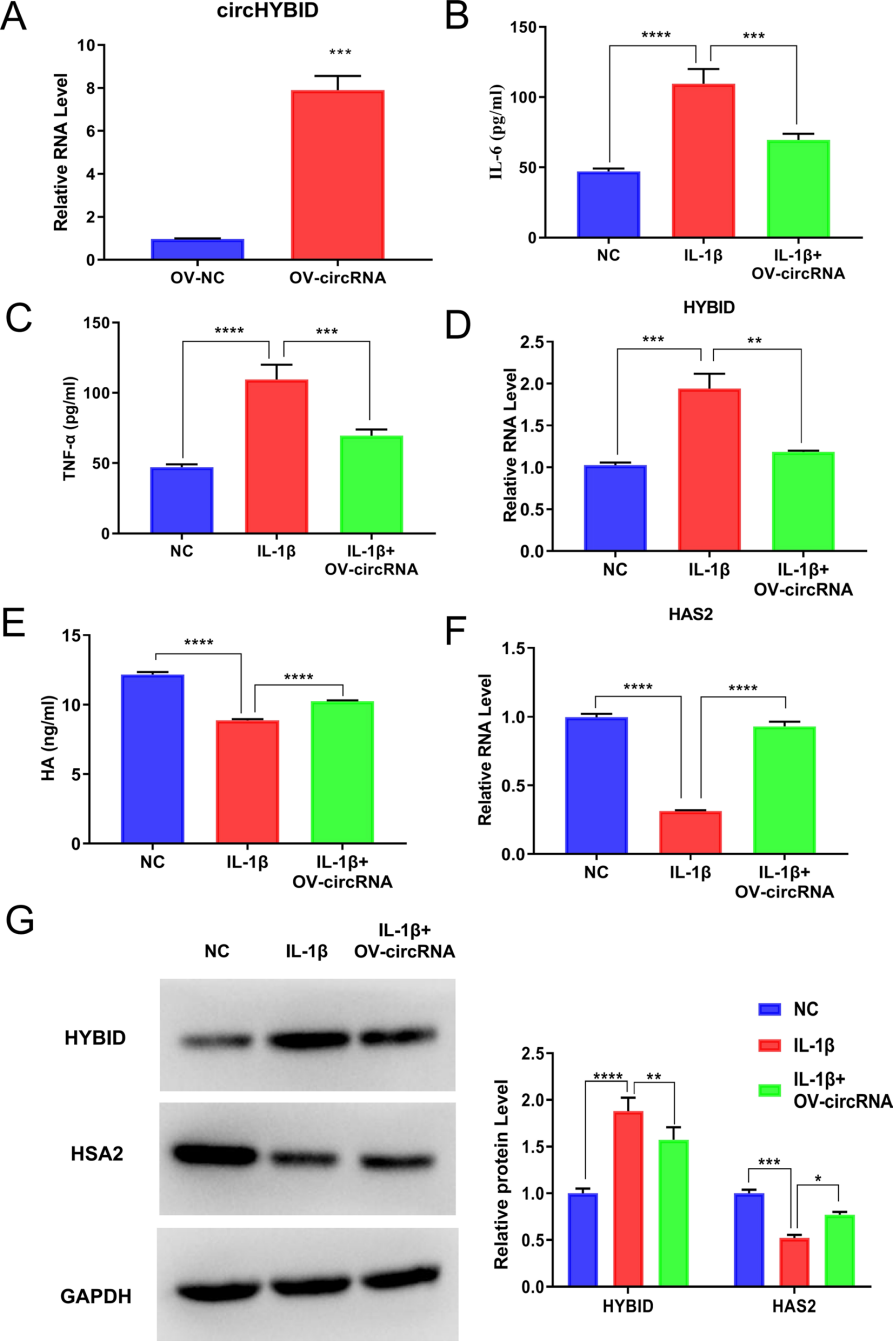
CircHYBID elevated hyaluronan (HA) accumulation. The expression of circHYBID (**A**), HYBID (**D**), and HAS2 (**F**) were assessed using quantitative real-time polymerase chain reaction in IL-1β-induced chondrocytes with or without overexpressed circHYBID. IL-6 (**B**), TNF-α (**C**), and HA (**E**) levels in cell culture supernatant were measured using enzyme-linked immunosorbent assay. **G** The protein expression of HAS2 and HYBID was detected using Western blotting. *p < 0.01; **p < 0.01; ***p < 0.001; ****p < 0.0001

To further verify the relationship and potential mechanism between circHYBID and HA metabolism, a circHYBID overexpression vector (OV-circRNA) was constructed, and vector LV003 was used as the negative control (OV-NC). They were transfected into chondrocytes, and the cells were collected and tested by qRT-PCR to verify the efficiency of circHYBID overexpression vector. The results showed that circHYBID expression was significantly increased in the OV-circRNA group compared with the OV-NC group, which indicated that circHYBID overexpression vector was successfully constructed (Fig. 5A). The overexpression of circHYBID partially reduced the inflammatory factors IL-6 and TNF-α’s expression in IL-1β-treated chondrocytes via the qRT-PCR assay (Fig. 5B and C). CircHYBID overexpression also decreased HYBID mRNA and protein expression in IL-1β-treated chondrocytes using qRT-PCR and WB analysis (Fig. 5D and G). In addition, circHYBID overexpression partially recovered HA in supernatant and HAS2 expression in chondrocytes with IL-1β stimulation by ELISA, qRT-PCR, and WB analyses (Fig. 5E–G). Based on the above results, we further confirmed that circHYBID overexpression increases HA accumulation after IL-1β treatment in primary chondrocytes by regulating the HYBID and HAS2 expression.

### CircHYBID regulates hsa-miR-29b-3p/TGF-β1 axis in OA

**Fig. 6:**
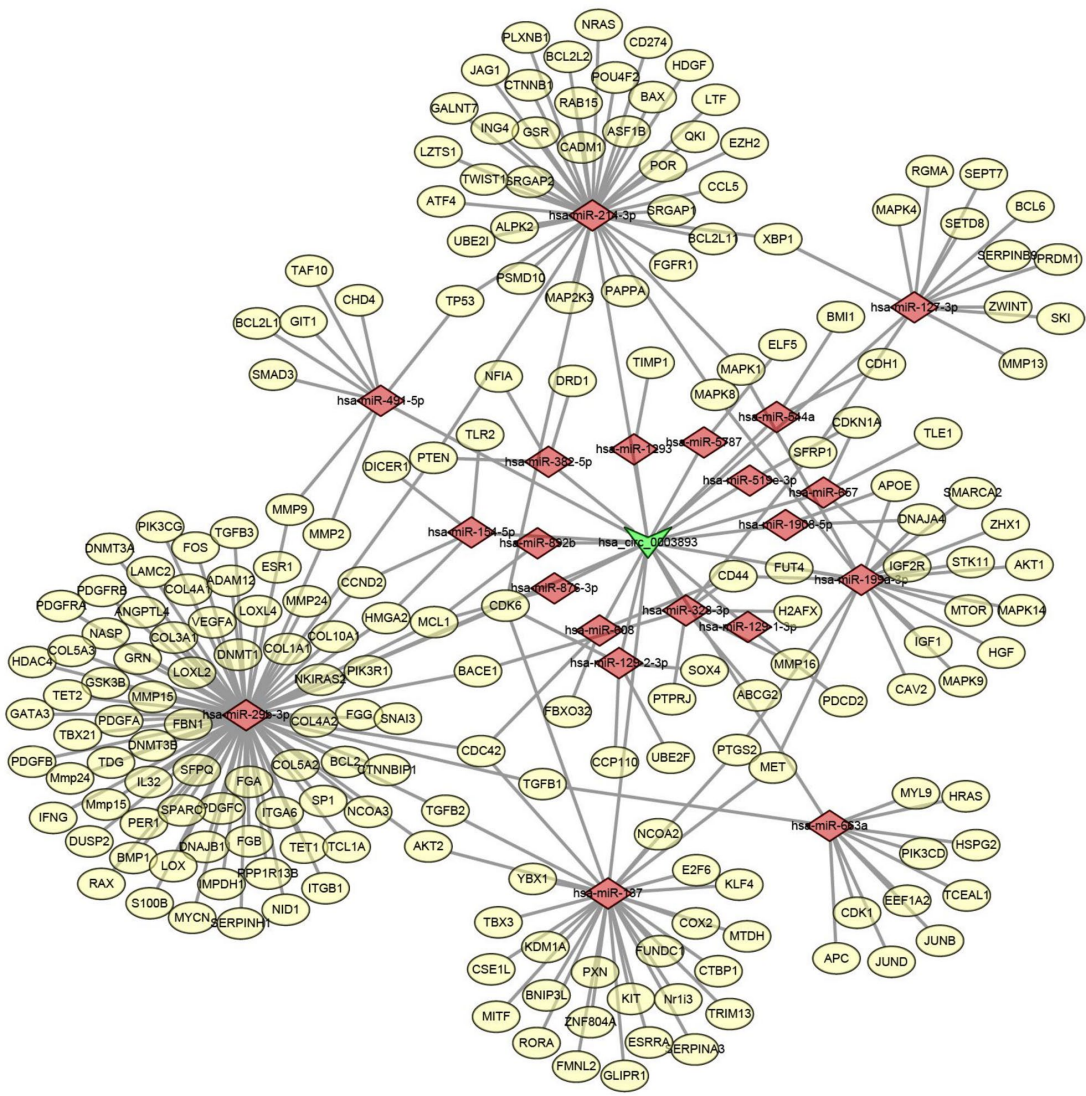
The circHYBID–miRNA–mRNA network. The oval represents mRNA, the rhombus denotes miRNA, the inverted triangle represents circRNA, and their relationship is depicted by one edge

**Fig. 7:**
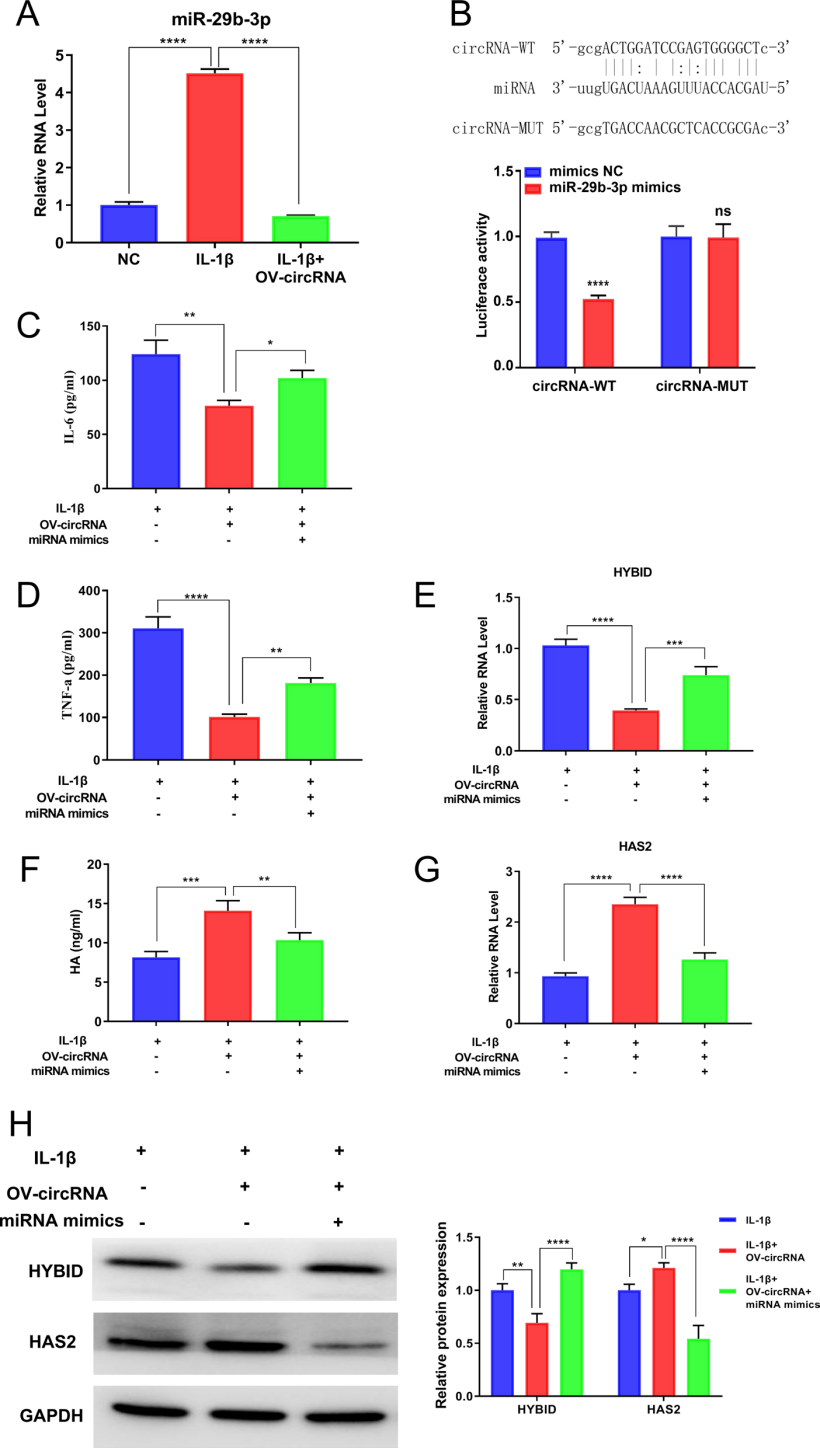
circHYBID acts as a sponge for hsa-miR-29b-3p in OA. **A** The expression of hsa-miR-29b-3p in IL-1β-induced chondrocytes with or without overexpressed circHYBID was analyzed using quantitative real-time polymerase chain reaction (PCR). **B** Dual-luciferase reporter assays confirmed the potential binding site of circHYBID and hsa-miR-29b-3p. IL-6 (**C**), TNF-α (**D**), and HA (**F**) levels in cell culture supernatant were measured using enzyme-linked immunosorbent assay in IL-1β-induced chondrocytes with overexpressed circHYBID or miR-29b-3p mimic treatment. The mRNA expressions of HYBID (**E**) and HAS2 (**G**) was assessed using quantitative real-time PCR. **H** The protein expressions of HAS2 and HYBID was detected using Western blotting. *p < 0.05; **p < 0.01; ***p < 0.001; ****p < 0.0001

As many circRNAs commonly function as miRNA sponges that competitively sequester and suppress miRNAs, then the circRNA–miRNA–mRNA network of circHYBID was established to explore the molecular mechanisms of circHYBID in OA (Fig. 6). Among the potential circHYBID’s downstream targets, hsa-miR-29b-3p caught our attention because it has been demonstrated to participate in OA progression (Le et al. 2016). Hsa-miR-29b-3p expression was detected using qRT-PCR, and the results show that hsa-miR-29b-3p expression was upregulated in chondrocytes treated with IL-1β. However, hsa-miR-29b-3p expression was inhibited by circHYBID overexpression in IL-1β-treated chondrocytes (Fig. 7A). This result indicated that hsa-miR-29b-3p may be a potential binding target miRNA for circHYBID. For further confirmation, wild type (circRNA-WT) and mutant type (circRNA-MUT) sequences of circHYBID binding to hsa-miR-29b-3p were cloned into dual-luciferase reporter vectors pmir-GLO. The aforementioned plasmids were then cotransfected into cells with hsa-miR-29b-3p mimics negative control (mimics NC) or hsa-miR-29b-3p mimics (miR-29b-3p mimics), and changes in luciferase activity were analyzed. Luciferase activity was significantly downregulated in cells cotransfected with circHYBID-WT and hsa-miR-29b-3p mimics, but no difference was observed between the circRNA-MUT+mimics NC and circRNA-MUT+miR-29b-3p mimics groups, indicating that hsa-miR-29b-3p maybe a potential binding target miRNA for circHYBID (Fig. 7B). To further verify whether circHYBID regulates miR-29b-3p in OA, we cotransfected circHYBID and miR-29b-3p mimics into chondrocytes and subsequently treated with IL-1β. qRT-PCR results indicated that miR-29b-3p mimics attenuated the inhibitory effect of circHYBID overexpression on inflammatory factors (IL-6 and TNF-α) and host gene HYBID expression under IL-1β treatment (Fig. 7C–E, and H). Moreover, they attenuated the promotion effect of circHYBID overexpression on HA in supernatant and HAS2 expression in chondrocytes under IL-1β treatment (Fig. 7F–H).

**Fig. 8:**
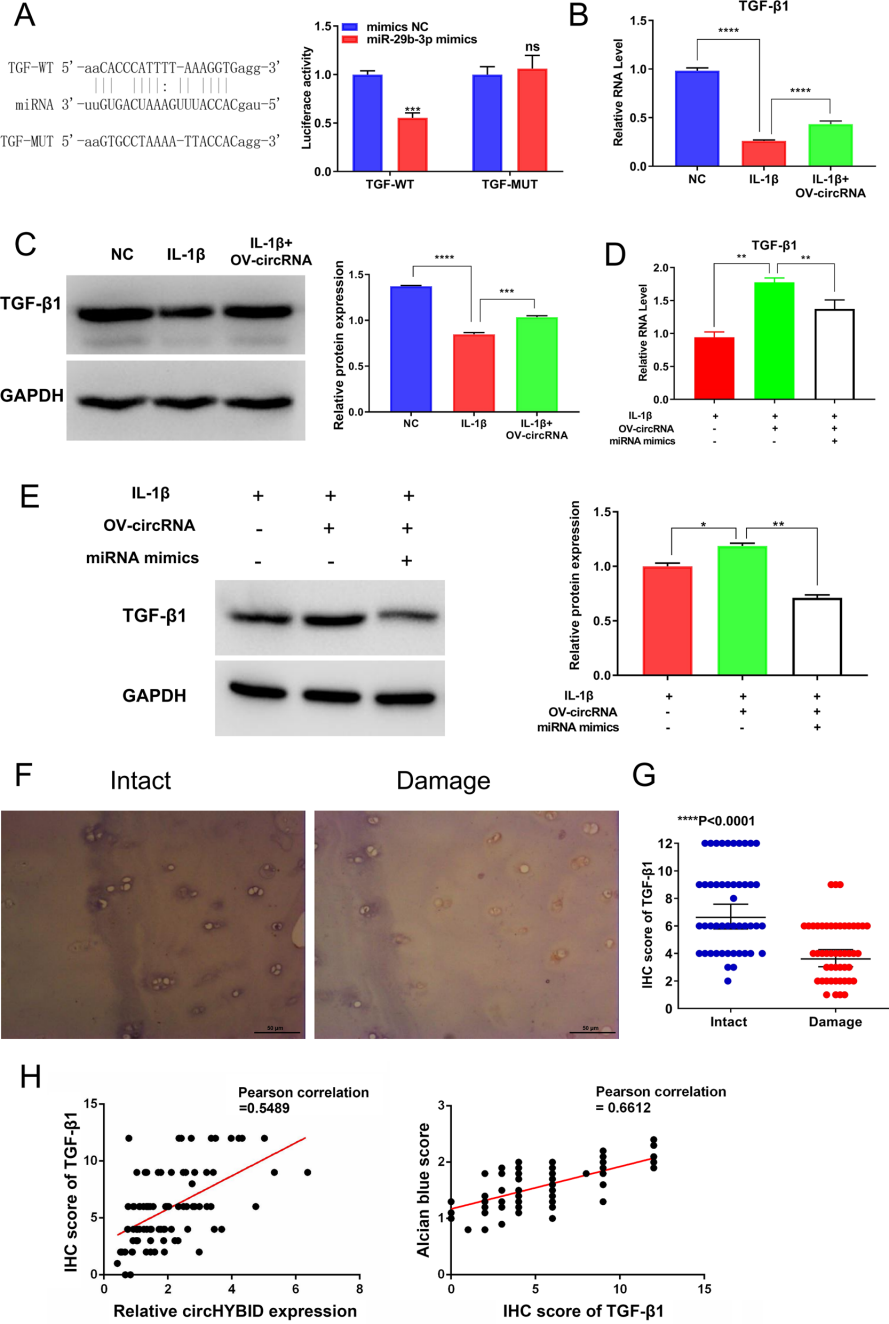
CircHYBID upregulates the expression of hsa-miR-29b-3p target gene—transforming growth factor (TGF)-β1. **A** Dual-luciferase reporter assays confirmed the potential binding site of TGF-β1 and hsa-miR-29b-3p. **B** The relative mRNA expression of TGF-β1 in IL-1β-induced chondrocytes with or without overexpressed circHYBID was analyzed using quantitative real-time polymerase chain reaction (PCR). **C** The protein expression of TGF-β1 in IL-1β-induced chondrocytes with or without overexpressed circHYBID was analyzed using Western blotting. **D** The relative mRNA expression of TGF-β1 in IL-1β-induced chondrocytes with overexpressed circHYBID or miR-29b-3p mimic treatment was analyzed using quantitative real-time PCR. **E** The protein expression of TGF-β1 in IL-1β-induced chondrocytes with overexpressed circHYBID or miR-29b-3p mimic treatment was analyzed using Western blotting. **F** The expression of TGF-β1 in paired samples of intact and damaged cartilage was analyzed using immunohistochemistry. Magnification: × 200, Scale bar = 50 μm. **G** IHC score of TGF-β1. **H** Correlation scatter plot of relative circHYBID expression and score of TGF-β1 (Pearson’s correlation, r = 0.5489), and correlation scatter plot of IHC score of TGF-β1 with alcian blue score (Pearson’s correlation, r = 0.6612). *p < 0.05; **p < 0.01; ***p < 0.001; ****p < 0.0001

From the circRNA–miRNA–mRNA network, we found that TGF-β1 was a potential downstream target of hsa-miR-29b-3p (Fig. 6). TGF-β1 has been reported to be closely related to HA metabolism and OA pathogenesis (Ongchai et al. 2018). In addition, a previous study has described the interaction between hsa-miR-29b-3p and TGF-β1 (Lu et al. 2017). Therefore, TGF-β1 was selected as the target mRNA for further research. The predicted binding site of miR-29b-3p and TGF-β1 is shown in Fig. 8A. The wild type and mutant type sequences of TGF-β1 (TGF-WT and TGF-MUT) were cloned into pmir-GLO for luciferase activity assay. The results showed that luciferase activity was significantly downregulated in TGF-WT+miR-29b-3p mimics compared with TGF-WT+mimics NC group, but no difference was observed between the TGF-MUT+mimics NC and TGF-MUT+miR-29b-3p mimics groups, indicating that TGF-β1 maybe a potential downstream target mRNA for hsa-miR-29b-3p (Fig. 8A). TGF-β1 mRNA and protein expression was then detected using qRT-PCR (Fig. 8B and D) and WB (Fig. 8C and E), respectively. The result illustrated that IL-1β inhibits the expression of TGF-β1 in chondrocytes, whereas circHYBID overexpression restored TGF-β1 expression, which was again inhibited by miR-29b-3p mimics (Fig. 8B–E). TGF-β1 expression was further verified in 48 pairs of cartilage samples using IHC. TGF-β1 expression was downregulated in damaged cartilage samples, and the median staining index of intact cartilage was 7.3 compared with 4.1 in damaged cartilage (Fig. 8F and G). Meanwhile, Pearson’s analyses indicated a positive correlation between relative circHYBID expression and scores of TGF-β1, between scores of alcian blue and scores of TGF-β1 in OA cartilage tissues (Fig. 8H). Altogether, these results revealed that circHYBID might regulate HA metabolism via the hsa-miR-29b-3p/TGF-β1 axis in OA.

## Discussion

OA is a common disabling joint disease, and no existing clinical options exist for preventing early OA from progressing to severe disease. To improve this situation, the underlying mechanisms of OA must be explored in detail for identifying strategies that promote the biological regeneration of cartilage tissue in the early stage of OA (Schinhan et al. 2020; Liao et al. 2021).

HA is a ubiquitous high-molecular-weight polymer comprising repeated disaccharides of glucuronic acid and *N*-acetylglucosamine, which is an important component of proteoglycan, and it is primarily found in the cartilage ECM between chondrocytes (Skandalis et al. 2020). HA constitutes ECM and stabilizes its integrity. It also participates in various biological processes, such as cellular and tissue development, migration, and repair after injury or inflammation, by binding to secretory proteins and cell surface receptors (Joy et al. 2018). The synthesis and degradation metabolism of HA are maintained by the dynamic regulation of key enzymes, such as CD44, HAS, hyaluronidase, and HYBID (Yoshida and Okada 2019; Zhai et al. 2020). HAS contributes to HA synthesis, and HAS isozymes include HAS1, HAS2, and HAS3. Among them, HAS1 and HAS2 synthesize high molecular weight HA, while HAS3 synthesize low molecular weight HA (Tammi et al. 2011). They have participate in different biological progress as its molecular weight: low molecular weight cause inflammation, high molecular weight is mainly synthesized in the joint, in which HAS2 is the main synthetase (Toole 2004). However, HYBID contributes to HA degradation (Yoshino et al. 2017). Dysregulated HA metabolism is an important factor in cartilage ECM degeneration and OA development. However, little is known regarding the functions of HA in the development and progression of OA. In the present study, alcian blue staining results using cartilage specimens from patients with OA revealed that proteoglycan of ECM and HA levels were lower in damaged cartilage specimens. Moreover, alcian blue score was inversely correlated with the Mankin score, indirectly suggesting that HA levels in cartilage maybe gradually decrease with OA progression. Then IL-1β was used to treat chondrocytes to construct OA model in vitro, and found that HA expression also dercreased after IL-1β treatment. HA synthetase, HAS2 expression was downregulated in chondrocytes treated with IL-1β, there was no significant difference in HAS1 and HAS3 expression under IL-1β stimulation, whereas HYBID expression was increased in chondrocytes treated with IL-1β. Thus, assessing the mechanism of HA metabolism in OA may provide a new perspective for clarifying the OA pathogenesis.

The role of circRNAs in the pathogenesis of OA has drawn increasing attention (Liu et al. 2017; Soslau 2018; Hansen et al. 2013). Studies have revealed that circRNAs play regulatory roles in cartilage ECM degradation, inflammatory response in chondrocytes, and apoptosis (Shen et al. 2020; Jiang et al. 2020). The present study investigated the pathogenesis of OA from the perspective of the regulation of HA metabolism by circRNAs. Among the identified differentially regulated circRNAs, circHYBID was identified as a key downregulated circRNA related to HA metabolism in OA cartilage for the first time.

CircHYBID is generated by the back splicing of the KIAA1199 gene, which comprises the protein coding sequence of HYBID mRNA. KIAA1199 has a key role in HA binding and depolymerization, and the encoded protein is also named HYBID or cell migration-inducing protein (Yoshida and Okada 2019). HYBID can catabolize HA via the clathrin-coated pit pathway and play an important role in ECM homeostasis. It is essential for the degradation of HA in skin, arthritic synovial fibroblasts, nucleus pulposus cells, and cartilage independently of HYAL1 and HYAL2/CD44. It is highly expressed by chondrocytes in the HA-depleted area of OA cartilage (Choi et al. 2019). In our study, we found that HYBID expression was upregulated in IL-1β-treated chondrocytes, HA in supernatant and HAS2 expression IL-1β-treated chondrocytes was downregulated. However, circHYBID overexpression decreased HYBID mRNA and protein expression in IL-1β-treated chondrocytes, partially recovered HA in supernatant and HAS2 expression in chondrocytes with IL-1β stimulation. These results indicated that circHYBID plays a protective role against OA progression.

Accumulating evidence supports the key regulatory role of the ceRNA mechanism in several diseases, including OA. The mechanistic ceRNA network of circHYBID was explored in this study. Our data indicated that TGF-β1 was a potential target for circHYBID. TGF-β1 was recently demonstrated to promote HA synthesis by regulating the expression of HAS2 (Ongchai et al. 2018). In particular, TGF-β1 has a stimulatory effect on equine chondrocytes, thereby enhancing HA synthesis and promoting ECM generation (Nagaoka et al. 2015; Ongchai et al. 2018). Shen et al. reported that CircCDK14 promotes Smad2 expression in the TGF-β signaling pathway, which protects against OA by maintaining chondrocyte ECM and regulating chondrocyte apoptosis and proliferation (Shen et al. 2020).

Based on our study findings, we propose a mechanism by which circHYBID acts as an hsa-miR-29b-3p sponge to upregulate TGF-β1 expression in the OA model. This axis promotes HA anabolism and suppresses HA catabolism, thereby increasing HA levels in cartilage.

## Conclusion

We identified the circHYBID–hsa-miR-29b-3p–TGF-β1 axis as a novel mechanism for HA metabolism in OA. CircHYBID overexpression elevated HA accumulation, suggesting its potential use as a target for preventing and treating OA. Deciphering the precise molecular mechanisms of circHYBID in OA is critical for understanding the pathogenesis of OA and for exploring new potential therapeutic targets.

## Supplementary Information

**Additional file 1: Table 1.** Primer sequences used in this study.

**Additional file 2: Table 2.** The Mankin score in cartilage samples.

**Additional file 3: Table 3.** Mankin score and alcian blue score of intact and damaged cartilage.

**Additional file 4: Table 4.** Samples for sequencing.

**Additional file 5: Table 5.** Differently expressed circRNAs between the NC and OA groups.

## Data Availability

The datasets used and/or analyzed during the current study are available from the corresponding author on reasonable request.

## Abbreviations

circRNA: Circular RNAs
CEMIP: Cell migration-inducing hyaluronan binding protein
ECM: Extracellular matrix
ELISA: Enzyme-linked immunosorbent assay
HA: Hyaluronan
HAS2: Hasynthase 2
HYAL: Hyaluronidase
HYBID: HA-binding protein involved in hyaluronan depolymerization
IHC: Immunohistochemistry
MUT: Mutated type
OA: Osteoarthritis
qRT-PCR: Quantitative real-time polymer chain reaction
TGF: Transforming growth factor
WB: Western blotting
WT: Wild-type
