# Supplementary material for: CircHYBID regulates hyaluronan metabolism in chondrocytes via hsa-miR-29b-3p/TGF-β1 axis
Source: Mol Med. 2021 May 31;27:56. doi: 10.1186/s10020-021-00319-x (PMC8165762; doi:10.1186/s10020-021-00319-x)
Supplement: Supplementary file 1 — Additional file 1: Table 1. Primer sequences used in this study. [file 10020_2021_319_MOESM1_ESM.docx]

Additional Table 1. Primer sequences used in this study.

| Name | Forward Sequence (5'-3') | Reverse Sequence (5'-3') |
| --- | --- | --- |
| IL-1β | TAGGGCTGGCAGAAAGGGAACA | GTGGGAGCGAATGACAGAGGGT |
| IL-6 | ACTCACCTCTTCAGAACGAATTG | CCATCTTTGGAAGGTTCAGGTTG |
| MMP-13 | GCAGTCTTTCTTCGGCTTAGAG | GTATTCACCCACATCAGGAACC |
| TNF-α | GCCAGAGGGCTGATTAGAGA | TCAGCCTCTTCTCCTTCCTG |
| HAS1 | CAAGATTCTTCAGTCTGGAC | TAAGAACGAGGAGAAAGCAG |
| HAS2 | CAGAATCCAAACAGACAGTTC | TAAGGTGTTGTGTGTGACTG |
| HYBID | TCCCAGACTAGCTACCACTCC | AGTGTGCTCCCTCTGGGTC |
| hsa_circ_0003893 | GGGACGAGGACTCAGGATGT | GTCCCAGTGGATGGTGTAGC |
| U6 | TGCGGGTGCTCGCTTCGGCAGC | CCAGTGCAGGGTCCGAGGT |
| GAPDH | GGGAAACTGTGGCGTGAT | GAGTGGGTGTCGCTGTTGA |
| circ_0003893-divergent | GGGACGAGGACTCAGGATGT | GTCCCAGTGGATGGTGTAGC |
| circ_0003893-convergent | TCCCAGACTAGCTACCACTCC | AGTGTGCTCCCTCTGGGTC |
| GAPDH-divergent | GAGTCAACGGATTTGGTCGT | GACAAGCTTCCCGTTCTCAG |
| GAPDH-convergent | CTGAGAACGGGAAGCTTGTC | ACGACCAAATCCGTTGACTC |
