# Supplementary material for: CircHYBID regulates hyaluronan metabolism in chondrocytes via hsa-miR-29b-3p/TGF-β1 axis
Source: Mol Med. 2021 May 31;27:56. doi: 10.1186/s10020-021-00319-x (PMC8165762; doi:10.1186/s10020-021-00319-x)
Supplement: Supplementary file 2 — Additional file 2: Table 2. The Mankin score in cartilage samples. [file 10020_2021_319_MOESM2_ESM.docx]

Additional Table 2. The Mankin score in cartilage samples.

| Patient | Gender | Age | Mankin score | |
| --- | --- | --- | --- | --- |
|  |  |  | Intact | Damage |
| 1 | Female | Y73 | 5 | 9 |
| 2 | Female | Y62 | 3 | 6 |
| 3 | Female | Y66 | 4 | 8 |
| 4 | Male | Y68 | 3 | 7 |
| 5 | Female | Y74 | 7 | 12 |
| 6 | Female | Y64 | 3 | 9 |
| 7 | Male | Y89 | 6 | 11 |
| 8 | Female | Y66 | 3 | 6 |
| 9 | Female | Y69 | 3 | 10 |
| 10 | Female | Y62 | 2 | 8 |
| 11 | Female | Y68 | 3 | 11 |
| 12 | Female | Y74 | 5 | 7 |
| 13 | Female | Y80 | 7 | 11 |
| 14 | Female | Y69 | 4 | 9 |
| 15 | Female | Y62 | 2 | 8 |
| 16 | Female | Y63 | 5 | 10 |
| 17 | Female | Y72 | 5 | 11 |
| 18 | Female | Y80 | 6 | 13 |
| 19 | Female | Y66 | 3 | 9 |
| 20 | Female | Y67 | 3 | 7 |
| 21 | Female | Y76 | 4 | 11 |
| 22 | Female | Y75 | 5 | 8 |
| 23 | Female | Y71 | 4 | 9 |
| 24 | Female | Y73 | 5 | 10 |
| 25 | Female | Y66 | 3 | 8 |
| 26 | Male | Y69 | 4 | 9 |
| 27 | Female | Y64 | 4 | 10 |
| 28 | Female | Y68 | 5 | 9 |
| 29 | Female | Y72 | 5 | 11 |
| 30 | Male | Y63 | 2 | 8 |
| 31 | Female | Y62 | 2 | 7 |
| 32 | Female | Y63 | 3 | 10 |
| 33 | Female | Y61 | 2 | 6 |
| 34 | Female | Y72 | 5 | 8 |
| 35 | Male | Y74 | 4 | 10 |
| 36 | Female | Y78 | 5 | 11 |
| 37 | Female | Y65 | 3 | 8 |
| 38 | Female | Y61 | 2 | 6 |
| 39 | Female | Y67 | 3 | 8 |
| 40 | Female | Y68 | 6 | 9 |
| 41 | Female | Y71 | 5 | 10 |
| 42 | Female | Y74 | 3 | 7 |
| 43 | Female | Y67 | 3 | 8 |
| 44 | Female | Y68 | 6 | 9 |
| 45 | Female | Y71 | 5 | 11 |
| 46 | Female | Y65 | 4 | 9 |
| 47 | Female | Y83 | 5 | 12 |
| 48 | Female | Y64 | 3 | 8 |
| mean | Female | Y64 | 4 | 9 |
