# Supplementary material for: CircHYBID regulates hyaluronan metabolism in chondrocytes via hsa-miR-29b-3p/TGF-β1 axis
Source: Mol Med. 2021 May 31;27:56. doi: 10.1186/s10020-021-00319-x (PMC8165762; doi:10.1186/s10020-021-00319-x)
Supplement: Supplementary file 3 — Additional file 3: Table 3. Mankin score and alcian blue score of intact and damaged cartilage. [file 10020_2021_319_MOESM3_ESM.docx]

Additional Table 3.Mankin score and alcian blue score of intact and damaged cartilage.

| Variable | Mankin score | Alcian blue score* |
| --- | --- | --- |
| Intact | 4 | 1.85 |
| Damaged | 9 | 1.35 |
| P-value# | <0.0001 | <0.0001 |

*The alcian blue score (ranging from 0 to 3) was calculated as the intensity of HA staining (0, negative; 1, weakly positive; 2, positive; 3, strongly positive). ^#^Wilcoxon’s signed-rank test, p < 0.0001.
