# Supplementary material for: CircHYBID regulates hyaluronan metabolism in chondrocytes via hsa-miR-29b-3p/TGF-β1 axis
Source: Mol Med. 2021 May 31;27:56. doi: 10.1186/s10020-021-00319-x (PMC8165762; doi:10.1186/s10020-021-00319-x)
Supplement: Supplementary file 4 — Additional file 4: Table 4. Samples for sequencing. [file 10020_2021_319_MOESM4_ESM.docx]

Additional Table 4. Samples for sequencing.

| Patient | Gender | Age | Mankin score | |
| --- | --- | --- | --- | --- |
|  |  |  | Intact | Damage |
| Patient 1 | Female | Y64 | 3 | 9 |
| Patient 2 | Female | Y68 | 3 | 11 |
| Patient 3 | Male | Y74 | 4 | 10 |
