# Supplementary material for: CircHYBID regulates hyaluronan metabolism in chondrocytes via hsa-miR-29b-3p/TGF-β1 axis
Source: Mol Med. 2021 May 31;27:56. doi: 10.1186/s10020-021-00319-x (PMC8165762; doi:10.1186/s10020-021-00319-x)
Supplement: Supplementary file 5 — Additional file 5: Table 5. Differently expressed circRNAs between the NC and OA groups. [file 10020_2021_319_MOESM5_ESM.docx]

Additional Table 5. Differently expressed circRNAs between the NC and OA groups.

| circRNA  _ID | circBase_ID | geneID | cho_ OA -Expression | cho_ NC- Expression | cho_ OA -TPM | cho_ NC -TPM | log2 Ratio (cho_OA /cho_NC) | Up-Down -Regulation (cho_NC /cho_OA) | P- value | FDR |
| --- | --- | --- | --- | --- | --- | --- | --- | --- | --- | --- |
| chr14:20811288 \|20811534 |  | RPPH1 | 0 | 111 | 0.001 | 6779.04 | 22.693 | Up | 9.86E-31 | 4.03E-27 |
| chr9:113734353 \|113735838 | hsa_circ_0087960 | LPAR1 | 0 | 71 | 0.001 | 4336.14 | 22.048 | Up | 6.57E-20 | 8.96E-17 |
| chr11:92085262 \|92088570 | hsa_circ_0000348 | FAT3 | 0 | 37 | 0.001 | 2259.68 | 21.108 | Up | 1.04E-10 | 4.26E-08 |
| chr17:75398141 \|75398785 | hsa_circ_0005320 | SEPT9 | 0 | 36 | 0.001 | 2198.61 | 21.068 | Up | 1.94E-10 | 7.23E-08 |
| chr2:189859772 \|189861222 |  | COL3A1 | 0 | 31 | 0.001 | 1893.25 | 20.852 | Up | 4.38E-09 | 1.19E-06 |
| chr10:31661947 \|31676195 | hsa_circ_0000228 | ZEB1 | 0 | 27 | 0.001 | 1648.96 | 20.653 | Up | 5.30E-08 | 1.27E-05 |
| chr16:68155890 \|68157024 | hsa_circ_0005615 | NFATC3 | 0 | 27 | 0.001 | 1648.96 | 20.653 | Up | 5.30E-08 | 1.20E-05 |
| chr15:64791492 \|64792365 | hsa_circ_0000615 | ZNF609 | 0 | 26 | 0.001 | 1587.88 | 20.599 | Up | 9.88E-08 | 2.02E-05 |
| chr15:81229015 \|81230320 | hsa_circ_0003893 | KIAA1199 | 0 | 20 | 0.001 | 1221.45 | 20.22 | Up | 4.15E-06 | 0.0007075 |
| chr2:231307652 \|231314970 | hsa_circ_0003922 | SP100 | 0 | 17 | 0.001 | 1038.23 | 19.986 | Up | 2.69E-05 | 0.0040773 |
| chr22:36694965 \|36696280 |  | MYH9 | 0 | 13 | 0.001 | 793.942 | 19.599 | Up | 0.00033 | 0.0391431 |
| chr1:14057495 \|14068652 | hsa_circ_0005986 | PRDM2 | 0 | 13 | 0.001 | 793.942 | 19.599 | Up | 0.00033 | 0.0380247 |
| chr1:117944808 \|117948267 | hsa_circ_0000116 | MAN1A2 | 0 | 13 | 0.001 | 793.942 | 19.599 | Up | 0.00033 | 0.0369685 |
| chr7:23015829 \|23030758 | hsa_circ_0005251 | FAM126A | 0 | 12 | 0.001 | 732.869 | 19.483 | Up | 0.00061 | 0.0653058 |
| chr9:138741983 \|138774924 | hsa_circ_0004338 | CAMSAP1 | 0 | 12 | 0.001 | 732.869 | 19.483 | Up | 0.00061 | 0.0636313 |
| chr2:100078958 \|100081447 | hsa_circ_0001053 | REV1 | 0 | 12 | 0.001 | 732.869 | 19.483 | Up | 0.00061 | 0.0620405 |
| chr17:77073512 \|77073946 | hsa_circ_0008114 | ENGASE | 0 | 12 | 0.001 | 732.869 | 19.483 | Up | 0.00061 | 0.0605273 |
| chr4:146767108 \|146770713 | hsa_circ_0003187 | ZNF827 | 0 | 10 | 0.001 | 610.724 | 19.22 | Up | 0.00211 | 0.1540811 |
| chr2:40655613 \|40657441 | hsa_circ_0005232 | SLC8A1 | 0 | 9 | 0.001 | 549.652 | 19.068 | Up | 0.00394 | 0.2681551 |
| chr11:1000432 \|1003804 | hsa_circ_0020749 | AP2A2 | 0 | 9 | 0.001 | 549.652 | 19.068 | Up | 0.00394 | 0.2637591 |
| chr10:114220283 \|114224416 | hsa_circ_0020045 | VTI1A | 0 | 9 | 0.001 | 549.652 | 19.068 | Up | 0.00394 | 0.2595049 |
| chr5:153413351 \|153414527 | hsa_circ_0001546 | FAM114A2 | 0 | 9 | 0.001 | 549.652 | 19.068 | Up | 0.00394 | 0.2553858 |
| chr21:47411924 \|47414143 |  | COL6A1 | 0 | 9 | 0.001 | 549.652 | 19.068 | Up | 0.00394 | 0.2513954 |
| chr2:36668401 \|36691798 | hsa_circ_0008966 | CRIM1 | 0 | 8 | 0.001 | 488.579 | 18.898 | Up | 0.00734 | 0.361459 |
| chr7:33976901 \|34014396 |  | BMPER | 0 | 8 | 0.001 | 488.579 | 18.898 | Up | 0.00734 | 0.3571559 |
| chr9:6420912 \|6434173 | hsa_circ_0002359 | UHRF2 | 0 | 8 | 0.001 | 488.579 | 18.898 | Up | 0.00734 | 0.3529541 |
| chr13:43368231 \|43372898 |  | snoU13, SNORA25 | 0 | 8 | 0.001 | 488.579 | 18.898 | Up | 0.00734 | 0.34885 |
| chr4:3088666 \|3109150 | hsa_circ_0001392 | HTT | 0 | 8 | 0.001 | 488.579 | 18.898 | Up | 0.00734 | 0.3448402 |
| chr18:9583115 \|9595100 | hsa_circ_0009022 | PPP4R1 | 0 | 8 | 0.001 | 488.579 | 18.898 | Up | 0.00734 | 0.3409215 |
| chr3:176665095 \|176700237 |  | snoU13,  Y_RNA, SNORA25,  U8 | 0 | 8 | 0.001 | 488.579 | 18.898 | Up | 0.00734 | 0.337091 |
| chr10:5815805 \|5842668 | hsa_circ_0017586 | GDI2 | 0 | 8 | 0.001 | 488.579 | 18.898 | Up | 0.00734 | 0.3333455 |
| chrX:54013511 \|54014377 | hsa_circ_0009069 | PHF8 | 0 | 8 | 0.001 | 488.579 | 18.898 | Up | 0.00734 | 0.3296824 |
| chr20:2967411 \|2969120 | hsa_circ_0001127 | PTPRA | 0 | 8 | 0.001 | 488.579 | 18.898 | Up | 0.00734 | 0.3260989 |
| chr9:86293356 \|86301070 | hsa_circ_0087357 | UBQLN1 | 0 | 8 | 0.001 | 488.579 | 18.898 | Up | 0.00734 | 0.3225924 |
| chr9:111812563 \|111812972 | hsa_circ_0087905 | TMEM245 | 0 | 8 | 0.001 | 488.579 | 18.898 | Up | 0.00734 | 0.3191606 |
| chr18:46858234 \|46906128 | hsa_circ_0002501 | DYM | 0 | 8 | 0.001 | 488.579 | 18.898 | Up | 0.00734 | 0.315801 |
| chr9:5968019 \|5988545 |  | KIAA2026 | 0 | 8 | 0.001 | 488.579 | 18.898 | Up | 0.00734 | 0.3125114 |
| chr12:69983265 \|69985939 | hsa_circ_0000418 | CCT2 | 0 | 7 | 0.001 | 427.507 | 18.706 | Up | 0.01368 | 0.4439834 |
| chr7:128231904 \|128249633 | hsa_circ_0006036 | RP11- 274B21.1 | 0 | 7 | 0.001 | 427.507 | 18.706 | Up | 0.01368 | 0.4404875 |
| chr4:5624264 \|5624718 |  | EVC2 | 0 | 7 | 0.001 | 427.507 | 18.706 | Up | 0.01368 | 0.4370462 |
| chr19:3425104 \|3435205 | hsa_circ_0006392 | NFIC | 0 | 7 | 0.001 | 427.507 | 18.706 | Up | 0.01368 | 0.4336582 |
| chr19:13135835 \|13136366 | hsa_circ_0005660 | NFIX | 0 | 7 | 0.001 | 427.507 | 18.706 | Up | 0.01368 | 0.4303224 |
| chr16:69404386 \|69406258 |  | TERF2 | 0 | 7 | 0.001 | 427.507 | 18.706 | Up | 0.01368 | 0.4270375 |
| chr7:69364272 \|69364484 | hsa_circ_0080414 | AUTS2 | 0 | 7 | 0.001 | 427.507 | 18.706 | Up | 0.01368 | 0.4238023 |
| chr15:94841430 \|94858866 |  | MCTP2 | 0 | 7 | 0.001 | 427.507 | 18.706 | Up | 0.01368 | 0.4206159 |
| chr6_qbl_hap6:1151791 \|1204098 |  | n/a | 0 | 7 | 0.001 | 427.507 | 18.706 | Up | 0.01368 | 0.4174769 |
| chr2:55894126 \|55895093 |  | PNPT1 | 0 | 7 | 0.001 | 427.507 | 18.706 | Up | 0.01368 | 0.4143845 |
| chr7:42187825 \|42188067 | hsa_circ_0008537 | GLI3 | 0 | 7 | 0.001 | 427.507 | 18.706 | Up | 0.01368 | 0.4113376 |
| chrX:64295354 \|64295792 | hsa_circ_0002532 | snoU13, Y_RNA,  U3 | 0 | 7 | 0.001 | 427.507 | 18.706 | Up | 0.01368 | 0.4083351 |
| chr15:89442638 \|89444890 |  | MFGE8 | 0 | 7 | 0.001 | 427.507 | 18.706 | Up | 0.01368 | 0.4053762 |
| chr22:46631030 \|46631406 | hsa_circ_0004706 | PPARA | 0 | 7 | 0.001 | 427.507 | 18.706 | Up | 0.01368 | 0.4024598 |
| chr15:59204762 \|59205895 | hsa_circ_0003713 | SLTM | 0 | 7 | 0.001 | 427.507 | 18.706 | Up | 0.01368 | 0.3995851 |
| chr11:66006275 \|66006748 | hsa_circ_0006857 | PACS1 | 0 | 7 | 0.001 | 427.507 | 18.706 | Up | 0.01368 | 0.3967511 |
| chr1:154145384 \|154145677 | hsa_circ_0008368 | TPM3 | 0 | 6 | 0.001 | 366.435 | 18.483 | Up | 0.02552 | 0.5113367 |
| chr11:66947550 \|66975159 | hsa_circ_0023052 | KDM2A | 0 | 6 | 0.001 | 366.435 | 18.483 | Up | 0.02552 | 0.5088423 |
| chr12:46285563 \|46287504 | hsa_circ_0004985 | ARID2 | 0 | 6 | 0.001 | 366.435 | 18.483 | Up | 0.02552 | 0.5063722 |
| chr4:128995615 \|129003460 | hsa_circ_0007619 | LARP1B | 0 | 6 | 0.001 | 366.435 | 18.483 | Up | 0.02552 | 0.503926 |
| chr15:76161292 \|76165909 | hsa_circ_0036376 | UBE2Q2 | 0 | 6 | 0.001 | 366.435 | 18.483 | Up | 0.02552 | 0.5015033 |
| chr11:70176279 \|70181800 |  | PPFIA1 | 0 | 6 | 0.001 | 366.435 | 18.483 | Up | 0.02552 | 0.4991037 |
| chr9:127670656 \|127674305 | hsa_circ_0002883 | GOLGA1 | 0 | 6 | 0.001 | 366.435 | 18.483 | Up | 0.02552 | 0.496727 |
| chr12:113705648 \|113707650 | hsa_circ_0002857 | TPCN1 | 0 | 6 | 0.001 | 366.435 | 18.483 | Up | 0.02552 | 0.4943729 |
| chr12:123062219 \|123065217 | hsa_circ_0004265 | KNTC1 | 0 | 6 | 0.001 | 366.435 | 18.483 | Up | 0.02552 | 0.4920409 |
| chr2:55535945 \|55536390 | hsa_circ_0054615 | CCDC88A | 0 | 6 | 0.001 | 366.435 | 18.483 | Up | 0.02552 | 0.4897309 |
| chr2:209165661 \|209167077 | hsa_circ_0057983 | PIKFYVE | 0 | 6 | 0.001 | 366.435 | 18.483 | Up | 0.02552 | 0.4874424 |
| chr6:56915572 \|56920595 |  | snoU13,  Y_RNA, U3,  SNORA40, SNORD112, SNORD45 | 0 | 6 | 0.001 | 366.435 | 18.483 | Up | 0.02552 | 0.4851752 |
| chr9:107513237 \|107521452 |  | NIPSNAP3A | 0 | 6 | 0.001 | 366.435 | 18.483 | Up | 0.02552 | 0.4829291 |
| chr1:9991949 \|9995685 | hsa_circ_0007803 | LZIC | 0 | 6 | 0.001 | 366.435 | 18.483 | Up | 0.02552 | 0.4807036 |
| chr16:83940592 \|83945972 |  | MLYCD | 0 | 6 | 0.001 | 366.435 | 18.483 | Up | 0.02552 | 0.4784985 |
| chr17:73038276 \|73038754 | hsa_circ_0006942 | ATP5H | 0 | 6 | 0.001 | 366.435 | 18.483 | Up | 0.02552 | 0.4763136 |
| chr11:581492 \|587464 | hsa_circ_0020670 | PHRF1 | 0 | 6 | 0.001 | 366.435 | 18.483 | Up | 0.02552 | 0.4741485 |
| chr6:161443042 \|161455481 |  | snoU13, SNORD45, MAP3K4 | 0 | 6 | 0.001 | 366.435 | 18.483 | Up | 0.02552 | 0.4720031 |
| chr16:74491772 \|74493687 | hsa_circ_0004315 | GLG1 | 0 | 6 | 0.001 | 366.435 | 18.483 | Up | 0.02552 | 0.4698769 |
| chr18:44526020 \|44526886 |  | KATNAL2 | 0 | 6 | 0.001 | 366.435 | 18.483 | Up | 0.02552 | 0.4677699 |
| chr3:71090479 \|71102924 | hsa_circ_0008234 | FOXP1 | 0 | 6 | 0.001 | 366.435 | 18.483 | Up | 0.02552 | 0.4656816 |
| chr9:33996221 \|34017187 | hsa_circ_0003410 | UBAP2 | 0 | 6 | 0.001 | 366.435 | 18.483 | Up | 0.02552 | 0.4636119 |
| chr4:3317797 \|3344780 | hsa_circ_0006091 | RGS12 | 0 | 6 | 0.001 | 366.435 | 18.483 | Up | 0.02552 | 0.4615605 |
| chr3:52734278 \|52734512 | hsa_circ_0066104 | GLT8D1 | 0 | 6 | 0.001 | 366.435 | 18.483 | Up | 0.02552 | 0.4595272 |
| chr1:113153463 \|113159517 | hsa_circ_0003277 | ST7L | 0 | 6 | 0.001 | 366.435 | 18.483 | Up | 0.02552 | 0.4575117 |
| chr12:2224390 \|2229596 | hsa_circ_0025016 | CACNA1C | 0 | 6 | 0.001 | 366.435 | 18.483 | Up | 0.02552 | 0.4555139 |
| chr20:35812583 \|35812776 | hsa_circ_0005759 | RPN2 | 0 | 6 | 0.001 | 366.435 | 18.483 | Up | 0.02552 | 0.4535334 |
| chr7:43400485 \|43404825 |  | snoU13,  Y_RNA,U3,  SNORA63, SNORA31, SNORA51,  HECW1 | 0 | 6 | 0.001 | 366.435 | 18.483 | Up | 0.02552 | 0.45157 |
| chr10:116730127 \|116734144 | hsa_circ_0020092 | TRUB1 | 0 | 6 | 0.001 | 366.435 | 18.483 | Up | 0.02552 | 0.4496236 |
| chr2:54119945 \|54120936 | hsa_circ_0054547 | PSME4 | 0 | 6 | 0.001 | 366.435 | 18.483 | Up | 0.02552 | 0.4476939 |
| chr21:38792601 \|38802702 | hsa_circ_0006062 | DYRK1A | 0 | 6 | 0.001 | 366.435 | 18.483 | Up | 0.02552 | 0.4457807 |
| chr19:13919650 \|13920034 | hsa_circ_0007836 | ZSWIM4 | 0 | 6 | 0.001 | 366.435 | 18.483 | Up | 0.02552 | 0.4438837 |
| chr3:129177442 \|129183624 |  | IFT122 | 0 | 6 | 0.001 | 366.435 | 18.483 | Up | 0.02552 | 0.4420029 |
| chr14:67812485 \|67814170 |  | ATP6V1D | 0 | 6 | 0.001 | 366.435 | 18.483 | Up | 0.02552 | 0.4401379 |
| chr7:151181823 \|151195266 | hsa_circ_0004779 | RHEB | 0 | 6 | 0.001 | 366.435 | 18.483 | Up | 0.02552 | 0.4382886 |
| chr2:144966170 \|144969146 | hsa_circ_0001072 | GTDC1 | 0 | 6 | 0.001 | 366.435 | 18.483 | Up | 0.02552 | 0.4364547 |
| chr17:60111148 \|60112969 | hsa_circ_0004273 | MED13 | 0 | 6 | 0.001 | 366.435 | 18.483 | Up | 0.02552 | 0.4346362 |
| chr10:95123718 \|95129533 | hsa_circ_0019201 | MYOF | 0 | 6 | 0.001 | 366.435 | 18.483 | Up | 0.02552 | 0.4328327 |
| chr6:42941741 \|42942776 | hsa_circ_0005038 | PEX6 | 0 | 6 | 0.001 | 366.435 | 18.483 | Up | 0.02552 | 0.4310441 |
| chr6:168944305 \|169008919 | hsa_circ_0078696 | SMOC2 | 0 | 6 | 0.001 | 366.435 | 18.483 | Up | 0.02552 | 0.4292703 |
| chr10:31644073 \|31750166 | hsa_circ_0008203 | ZEB1 | 0 | 6 | 0.001 | 366.435 | 18.483 | Up | 0.02552 | 0.427511 |
| chr18:55398859 \|55399061 | hsa_circ_0008105 | ATP8B1 | 0 | 6 | 0.001 | 366.435 | 18.483 | Up | 0.02552 | 0.425766 |
| chr7:33028119 \|33028264 | hsa_circ_0006227 | FKBP9 | 0 | 6 | 0.001 | 366.435 | 18.483 | Up | 0.02552 | 0.4240353 |
| chr2:135010666 \|135012215 | hsa_circ_0001068 | MGAT5 | 0 | 5 | 0.001 | 305.362 | 18.22 | Up | 0.04758 | 0.5105215 |
| chr2:227771509 \|227779067 | hsa_circ_0058497 | RHBDD1 | 0 | 5 | 0.001 | 305.362 | 18.22 | Up | 0.04758 | 0.509185 |
| chr15:62325640 \|62336454 | hsa_circ_0005761 | VPS13C | 0 | 5 | 0.001 | 305.362 | 18.22 | Up | 0.04758 | 0.5078555 |
| chr8:124089351 \|124117704 | hsa_circ_0085439 | TBC1D31 | 0 | 5 | 0.001 | 305.362 | 18.22 | Up | 0.04758 | 0.506533 |
| chr11:77386081 \|77389198 |  | RSF1 | 0 | 5 | 0.001 | 305.362 | 18.22 | Up | 0.04758 | 0.5052173 |
| chr17:43197692 \|43198739 | hsa_circ_0044158 | PLCD3 | 0 | 5 | 0.001 | 305.362 | 18.22 | Up | 0.04758 | 0.5039085 |
| chr13:76301165 \|76335174 | hsa_circ_0007610 | RP11-29 G8.3, LMO7 | 0 | 5 | 0.001 | 305.362 | 18.22 | Up | 0.04758 | 0.5026064 |
| chr8:42183488 \|42186732 | hsa_circ_0001793 | IKBKB | 0 | 5 | 0.001 | 305.362 | 18.22 | Up | 0.04758 | 0.501311 |
| chr3:31617888 \|31621588 | hsa_circ_0001278 | STT3B | 0 | 5 | 0.001 | 305.362 | 18.22 | Up | 0.04758 | 0.5000223 |
| chr4:106367540 \|106377902 | hsa_circ_0007477 | PPA2 | 0 | 5 | 0.001 | 305.362 | 18.22 | Up | 0.04758 | 0.4987402 |
| chr2:131796426 \|131801156 | hsa_circ_0056473 | ARHGEF4 | 0 | 5 | 0.001 | 305.362 | 18.22 | Up | 0.04758 | 0.4974646 |
| chr10:12571821 \|12595355 |  | Y_RNA, snoU13, CAMK1D | 0 | 5 | 0.001 | 305.362 | 18.22 | Up | 0.04758 | 0.4961956 |
| chr1:215342542 \|215345526 | hsa_circ_0008788 | KCNK2 | 0 | 5 | 0.001 | 305.362 | 18.22 | Up | 0.04758 | 0.494933 |
| chr6:160103506 \|160106065 | hsa_circ_0005472 | SOD2 | 0 | 5 | 0.001 | 305.362 | 18.22 | Up | 0.04758 | 0.4936768 |
| chr15:63845914 \|63855207 | hsa_circ_0008153 | USP3 | 0 | 5 | 0.001 | 305.362 | 18.22 | Up | 0.04758 | 0.492427 |
| chr5:140961879 \|140963182 | hsa_circ_0008982 | DIAPH1 | 0 | 5 | 0.001 | 305.362 | 18.22 | Up | 0.04758 | 0.4911835 |
| chrX:117676688 \|117680079 |  | DOCK11 | 0 | 5 | 0.001 | 305.362 | 18.22 | Up | 0.04758 | 0.4899463 |
| chr1:171537386 \|171544267 | hsa_circ_0004412 | PRRC2C | 0 | 5 | 0.001 | 305.362 | 18.22 | Up | 0.04758 | 0.4887153 |
| chr10:101659676 \|101659823 | hsa_circ_0005796 | DNMBP | 0 | 5 | 0.001 | 305.362 | 18.22 | Up | 0.04758 | 0.4874904 |
| chr4:17503342 \|17510986 | hsa_circ_0069249 | QDPR | 0 | 5 | 0.001 | 305.362 | 18.22 | Up | 0.04758 | 0.4862717 |
| chr7:133002038 \|133059756 | hsa_circ_0008419 | EXOC4 | 0 | 5 | 0.001 | 305.362 | 18.22 | Up | 0.04758 | 0.485059 |
| chr12:32458631 \|32459056 | hsa_circ_0025839 | BICD1 | 0 | 5 | 0.001 | 305.362 | 18.22 | Up | 0.04758 | 0.4838524 |
| chr11:61205097 \|61205585 | hsa_circ_0000311 | RP11-286 N22.8, SDHAF2 | 0 | 5 | 0.001 | 305.362 | 18.22 | Up | 0.04758 | 0.4826518 |
| chr11:85707869 \|85742653 | hsa_circ_0023923 | PICALM | 0 | 5 | 0.001 | 305.362 | 18.22 | Up | 0.04758 | 0.4814571 |
| chr21:37619815 \|37620866 | hsa_circ_0001187 | DOPEY2 | 0 | 5 | 0.001 | 305.362 | 18.22 | Up | 0.04758 | 0.4802683 |
| chr8:141840571 \|141889736 |  | PTK2 | 0 | 5 | 0.001 | 305.362 | 18.22 | Up | 0.04758 | 0.4790854 |
| chr11:120276827 \|120280159 | hsa_circ_0024605 | ARHGEF12 | 0 | 5 | 0.001 | 305.362 | 18.22 | Up | 0.04758 | 0.4779083 |
| chr1:219366424 \|219414650 | hsa_circ_0004417 | snoU13,  Y_RNA, SNORD  112, U3, SNORA51, SNORA25, SNORA72 | 0 | 5 | 0.001 | 305.362 | 18.22 | Up | 0.04758 | 0.4767369 |
| chr8:42761316 \|42785315 | hsa_circ_0003078 | HOOK3 | 0 | 5 | 0.001 | 305.362 | 18.22 | Up | 0.04758 | 0.4755713 |
| chr6:144086398 \|144128270 | hsa_circ_0078059 | PHACTR2 | 0 | 5 | 0.001 | 305.362 | 18.22 | Up | 0.04758 | 0.4744114 |
| chr7:72883847 \|72884813 | hsa_circ_0003866 | BAZ1B | 0 | 5 | 0.001 | 305.362 | 18.22 | Up | 0.04758 | 0.4732571 |
| chr3:29910349 \|29941246 | hsa_circ_0064644 | RBMS3 | 0 | 5 | 0.001 | 305.362 | 18.22 | Up | 0.04758 | 0.4721084 |
| chr5:97004255 \|97006388 | hsa_circ_0003213 | RP11-1E3.1 | 0 | 5 | 0.001 | 305.362 | 18.22 | Up | 0.04758 | 0.4709653 |
| chr11:66526514 \|66529921 |  | C11orf80 | 0 | 5 | 0.001 | 305.362 | 18.22 | Up | 0.04758 | 0.4698277 |
| chr9:4823548 \|4833228 | hsa_circ_0006134 | RCL1 | 0 | 5 | 0.001 | 305.362 | 18.22 | Up | 0.04758 | 0.4686956 |
| chr17:3969741 \|3976050 | hsa_circ_0004767 | ZZEF1 | 0 | 5 | 0.001 | 305.362 | 18.22 | Up | 0.04758 | 0.4675689 |
| chr19:40964062 \|40964452 | hsa_circ_0003061 | BLVRB | 0 | 5 | 0.001 | 305.362 | 18.22 | Up | 0.04758 | 0.4664477 |
| chr7:139741444 \|139757834 | hsa_circ_0004684 | PARP12 | 0 | 5 | 0.001 | 305.362 | 18.22 | Up | 0.04758 | 0.4653318 |
| chr5:176618885 \|176631293 | hsa_circ_0075157 | NSD1 | 0 | 5 | 0.001 | 305.362 | 18.22 | Up | 0.04758 | 0.4642212 |
| chr18:43490486 \|43492421 | hsa_circ_0047528 | EPG5 | 0 | 5 | 0.001 | 305.362 | 18.22 | Up | 0.04758 | 0.4631159 |
| chr19:12360793 \|12361183 | hsa_circ_0007222 | ZNF44 | 0 | 5 | 0.001 | 305.362 | 18.22 | Up | 0.04758 | 0.4620159 |
| chr20:36668837 \|36694658 | hsa_circ_0008006 | RPRD1B | 0 | 5 | 0.001 | 305.362 | 18.22 | Up | 0.04758 | 0.460921 |
| chr19:33390763 \|33409245 |  | CEP89 | 0 | 5 | 0.001 | 305.362 | 18.22 | Up | 0.04758 | 0.4598314 |
| chr16:88008654 \|88017865 | hsa_circ_0040809 | BANP | 0 | 5 | 0.001 | 305.362 | 18.22 | Up | 0.04758 | 0.4587469 |
| chr4:56269403 \|56284152 |  | TMEM165 | 0 | 5 | 0.001 | 305.362 | 18.22 | Up | 0.04758 | 0.4576675 |
| chr16:11988811 \|11991892 | hsa_circ_0037911 | GSPT1 | 0 | 5 | 0.001 | 305.362 | 18.22 | Up | 0.04758 | 0.4565931 |
| chr1:16263658 \|16264501 | hsa_circ_0010117 | SPEN | 0 | 5 | 0.001 | 305.362 | 18.22 | Up | 0.04758 | 0.4555238 |
| chr3:27385763 \|27394410 | hsa_circ_0003925 | NEK10 | 0 | 5 | 0.001 | 305.362 | 18.22 | Up | 0.04758 | 0.4544595 |
| chr12:124911168 \|124922542 | hsa_circ_0029311 | NCOR2 | 0 | 5 | 0.001 | 305.362 | 18.22 | Up | 0.04758 | 0.4534002 |
| chr17:17787948 \|17797124 | hsa_circ_0042265 | TOM1L2 | 0 | 5 | 0.001 | 305.362 | 18.22 | Up | 0.04758 | 0.4523458 |
| chr3:171965323 \|171979873 |  | snoU13,  Y_RNA, SNORA25, U8, SNORA72,  FNDC3B | 0 | 5 | 0.001 | 305.362 | 18.22 | Up | 0.04758 | 0.4512962 |
| chr4:170428188 \|170459062 |  | NEK1 | 0 | 5 | 0.001 | 305.362 | 18.22 | Up | 0.04758 | 0.4502516 |
| chr4:110580167 \|110585550 | hsa_circ_0070660 | CCDC109B | 0 | 5 | 0.001 | 305.362 | 18.22 | Up | 0.04758 | 0.4492117 |
| chr15:77656416 \|77657567 |  | snoU13,  Y_RNA, SNORD112, PEAK1 | 0 | 5 | 0.001 | 305.362 | 18.22 | Up | 0.04758 | 0.4481767 |
| chr1:246078832 \|246093239 | hsa_circ_0005090 | SMYD3 | 0 | 5 | 0.001 | 305.362 | 18.22 | Up | 0.04758 | 0.4471464 |
| chr13:21742127 \|21742538 | hsa_circ_0000467 | SKA3 | 0 | 5 | 0.001 | 305.362 | 18.22 | Up | 0.04758 | 0.4461208 |
| chr4:40892381 \|40895428 | hsa_circ_0069559 | APBB2 | 0 | 5 | 0.001 | 305.362 | 18.22 | Up | 0.04758 | 0.4450999 |
| chr2:191774989 \|191778090 | hsa_circ_0002428 | GLS | 0 | 5 | 0.001 | 305.362 | 18.22 | Up | 0.04758 | 0.4440837 |
| chr14:39627489 \|39628754 | hsa_circ_0002395 | TRAPPC6B | 0 | 5 | 0.001 | 305.362 | 18.22 | Up | 0.04758 | 0.4430722 |
| chr1:222897434 \|222898897 | hsa_circ_0003413 | BROX | 0 | 5 | 0.001 | 305.362 | 18.22 | Up | 0.04758 | 0.4420652 |
| chr3:196842798 \|196846401 | hsa_circ_0001383 | DLG1 | 0 | 5 | 0.001 | 305.362 | 18.22 | Up | 0.04758 | 0.4410628 |
| chr2:29356521 \|29368233 | hsa_circ_0006908 | CLIP4 | 0 | 5 | 0.001 | 305.362 | 18.22 | Up | 0.04758 | 0.4400649 |
| chr1:220179448 \|220180680 | hsa_circ_0007739 | EPRS | 0 | 5 | 0.001 | 305.362 | 18.22 | Up | 0.04758 | 0.4390715 |
| chr19:20828490 \|20829211 | hsa_circ_0050277 | ZNF626, CTC-513 N18.7 | 0 | 5 | 0.001 | 305.362 | 18.22 | Up | 0.04758 | 0.4380826 |
| chr3:123512524 \|123595464 | hsa_circ_0067063 | MYLK | 0 | 5 | 0.001 | 305.362 | 18.22 | Up | 0.04758 | 0.4370981 |
| chr1:213251038 \|213290752 | hsa_circ_0004849 | RPS6KC1 | 0 | 5 | 0.001 | 305.362 | 18.22 | Up | 0.04758 | 0.4361181 |
| chr18:55833020 \|55919286 |  | NEDD4L | 0 | 5 | 0.001 | 305.362 | 18.22 | Up | 0.04758 | 0.4351425 |
| chr3:122842913 \|122843190 | hsa_circ_0067029 | PDIA5 | 0 | 5 | 0.001 | 305.362 | 18.22 | Up | 0.04758 | 0.4341712 |
| chr5:167927596 \|167929110 | hsa_circ_0007958 | RARS | 0 | 5 | 0.001 | 305.362 | 18.22 | Up | 0.04758 | 0.4332042 |
| chr15:50741597 \|50751359 | hsa_circ_0035214 | USP8 | 0 | 5 | 0.001 | 305.362 | 18.22 | Up | 0.04758 | 0.4322415 |
| chr3:128514203 \|128516912 | hsa_circ_0067233 | RAB7A | 0 | 5 | 0.001 | 305.362 | 18.22 | Up | 0.04758 | 0.4312831 |
| chr20:34243124 \|34246936 | hsa_circ_0005587 | RP1-309 K20.6,  CPNE1 | 0 | 5 | 0.001 | 305.362 | 18.22 | Up | 0.04758 | 0.4303289 |
| chr7:35707044 \|35712888 | hsa_circ_0001696 | HERPUD2 | 0 | 5 | 0.001 | 305.362 | 18.22 | Up | 0.04758 | 0.429379 |
| chr22:38083917 \|38084999 | hsa_circ_0004543 | NOL12 | 0 | 5 | 0.001 | 305.362 | 18.22 | Up | 0.04758 | 0.4284332 |
| chr18:10855351 \|10979658 |  | PIEZO2 | 0 | 5 | 0.001 | 305.362 | 18.22 | Up | 0.04758 | 0.4274916 |
| chr1:92798948 \|92846430 | hsa_circ_0000091 | RPAP2 | 0 | 5 | 0.001 | 305.362 | 18.22 | Up | 0.04758 | 0.4265541 |
| chr6:74432974 \|74446231 | hsa_circ_0002465 | CD109 | 0 | 5 | 0.001 | 305.362 | 18.22 | Up | 0.04758 | 0.4256207 |
| chr7:6854395 \|6862991 | hsa_circ_0003943 | CCZ1B | 0 | 5 | 0.001 | 305.362 | 18.22 | Up | 0.04758 | 0.4246914 |
| chr16:354304 \|364683 | hsa_circ_0037158 | AXIN1 | 0 | 5 | 0.001 | 305.362 | 18.22 | Up | 0.04758 | 0.4237662 |
| chr1:156100418 \|156104766 |  | LMNA | 0 | 5 | 0.001 | 305.362 | 18.22 | Up | 0.04758 | 0.4228449 |
| chr13:42385361 \|42393522 | hsa_circ_0004711 | VWA8 | 0 | 5 | 0.001 | 305.362 | 18.22 | Up | 0.04758 | 0.4219277 |
| chr10:78771725 \|78787649 | hsa_circ_0005967 | KCNMA1 | 0 | 5 | 0.001 | 305.362 | 18.22 | Up | 0.04758 | 0.4210144 |
| chr15:100871094 \|100874385 |  | snoU13, Y_RNA, ADAMTS17 | 0 | 5 | 0.001 | 305.362 | 18.22 | Up | 0.04758 | 0.4201051 |
| chr6:26056054 \|26056248 |  | HIST1H1C | 0 | 5 | 0.001 | 305.362 | 18.22 | Up | 0.04758 | 0.4191997 |
| chr17:47388674 \|47389404 | hsa_circ_0003258 | ZNF652 | 2 | 12 | 141.26 | 732.869 | 2.375 | Up | 0.01614 | 0.4583358 |
| chr9:112898407 \|112900819 | hsa_circ_0008706 | PALM2- AKAP2, AKAP2 | 2 | 12 | 141.26 | 732.869 | 2.375 | Up | 0.01614 | 0.4551748 |
| chr7:32852369 \|32860353 | hsa_circ_0005885 | DPY19L1P2 | 2 | 12 | 141.26 | 732.869 | 2.375 | Up | 0.01614 | 0.4520572 |
| chr9:33351558 \|33352717 | hsa_circ_0005405 | NFX1 | 2 | 11 | 141.26 | 671.797 | 2.25 | Up | 0.02642 | 0.4354346 |
| chr7:77210744 \|77221573 | hsa_circ_0002458 | PTPN12 | 3 | 14 | 211.89 | 855.014 | 2.013 | Up | 0.01773 | 0.4897822 |
| chr1:16044388 \|16047883 | hsa_circ_0000021 | PLEKHM2 | 4 | 18 | 282.53 | 1099.3 | 1.96 | Up | 0.00753 | 0.3141678 |
| chr14:39746138 \|39748741 | hsa_circ_0000530 | RP11-407 N17.3, CTAGE5 | 3 | 13 | 211.89 | 793.942 | 1.906 | Up | 0.02797 | 0.4573262 |
| chr4:39739040 \|39757359 | hsa_circ_0009154 | UBE2K | 3 | 13 | 211.89 | 793.942 | 1.906 | Up | 0.02797 | 0.4555042 |
| chr3:196533450 \|196534785 | hsa_circ_0003036 | PAK2 | 3 | 12 | 211.89 | 732.869 | 1.79 | Up | 0.04369 | 0.4724517 |
| chr3:66286968 \|66313803 | hsa_circ_0003101 | SLC25A26 | 3 | 12 | 211.89 | 732.869 | 1.79 | Up | 0.04369 | 0.4712051 |
| chr2:202010101 \|202014558 | hsa_circ_0001092 | CFLAR | 4 | 14 | 282.53 | 855.014 | 1.598 | Up | 0.04243 | 0.6595794 |
| chr9:37424842 \|37426651 | hsa_circ_0001861 | GRHPR | 10 | 34 | 706.31 | 2076.46 | 1.556 | Up | 0.00143 | 0.1241098 |
| chr15:76152219 \|76165909 |  | UBE2Q2 | 8 | 25 | 565.05 | 1526.81 | 1.434 | Up | 0.01052 | 0.352466 |
| chr7:65751498 \|65751696 | hsa_circ_0006365 | TPST1 | 7 | 21 | 494.42 | 1282.52 | 1.375 | Up | 0.02376 | 0.4783766 |
| chr4:177632653 \|177650900 | hsa_circ_0004496 | VEGFC | 8 | 23 | 565.05 | 1404.67 | 1.314 | Up | 0.02193 | 0.4482042 |
| chr3:63898264 \|63898901 | hsa_circ_0007761 | ATXN7 | 9 | 24 | 635.68 | 1465.74 | 1.205 | Up | 0.02838 | 0.4604418 |
| chr10:116879949 \|116889297 | hsa_circ_0020093 | ATRNL1 | 8 | 21 | 565.05 | 1282.52 | 1.183 | Up | 0.04419 | 0.4754387 |
| chr14:73614503 \|73614802 | hsa_circ_0008521 | PSEN1 | 9 | 23 | 635.68 | 1404.67 | 1.144 | Up | 0.03974 | 0.6200872 |
| chr9:138773479 \|138774924 | hsa_circ_0001900 | CAMSAP1 | 20 | 50 | 1412.6 | 3053.62 | 1.112 | Up | 0.00262 | 0.1846197 |
| chr9:134381501 \|134381840 | hsa_circ_0001897 | POMT1 | 16 | 40 | 1130.1 | 2442.9 | 1.112 | Up | 0.0073 | 0.363821 |
| chr1:180953813 \|180962561 | hsa_circ_0007905 | STX6 | 23 | 13 | 1624.5 | 793.942 | -1.033 | Down | 0.03631 | 0.5753591 |
| chr4:153332455 \|153333681 | hsa_circ_0001451 | FBXW7 | 44 | 22 | 3107.8 | 1343.59 | -1.21 | Down | 0.00096 | 0.0852625 |
| chr3:157839892 \|157841780 | hsa_circ_0001355 | RSRC1 | 19 | 9 | 1342 | 549.652 | -1.288 | Down | 0.0237 | 0.4795507 |
| chrX:102094729 \|102160665 |  | snoU13,Y_RNA, U3, 5S_rRNA | 15 | 7 | 1059.5 | 427.507 | -1.309 | Down | 0.0425 | 0.6581339 |
| chr7:90355881 \|90356126 | hsa_circ_0001721 | CDK14 | 15 | 7 | 1059.5 | 427.507 | -1.309 | Down | 0.0425 | 0.6556504 |
| chr4:37633007 \|37640126 | hsa_circ_0001400 | RELL1 | 47 | 21 | 3319.7 | 1282.52 | -1.372 | Down | 0.00017 | 0.0212789 |
| chr1:151139410 \|151139890 | hsa_circ_0000128 | SCNM1 | 19 | 8 | 1342 | 488.579 | -1.458 | Down | 0.01298 | 0.4246542 |
| chr6:163876311 \|163899928 | hsa_circ_0005328 | QKI | 12 | 5 | 847.58 | 305.362 | -1.473 | Down | 0.04853 | 0.4266342 |
| chr2:233612325 \|233626146 | hsa_circ_0003341 | GIGYF2 | 17 | 7 | 1200.7 | 427.507 | -1.49 | Down | 0.01713 | 0.4764211 |
| chr1:44386076 \|44386600 | hsa_circ_0006595 | ST3GAL3 | 11 | 4 | 776.95 | 244.29 | -1.669 | Down | 0.03933 | 0.6159473 |
| chr16:67662273 \|67663436 | hsa_circ_0008272 | CTCF | 14 | 5 | 988.84 | 305.362 | -1.695 | Down | 0.01808 | 0.4928329 |
| chr5:64747302 \|64769779 | hsa_circ_0072688 | ADAMTS6 | 20 | 7 | 1412.6 | 427.507 | -1.724 | Down | 0.004 | 0.2513768 |
| chr16:68155890 \|68160513 | hsa_circ_0000711 | NFATC3 | 50 | 17 | 3531.6 | 1038.23 | -1.766 | Down | 2.96E-06 | 0.0005266 |
| chr1:203151859 \|203152919 |  | CHI3L1 | 12 | 4 | 847.58 | 244.29 | -1.795 | Down | 0.02336 | 0.4751924 |
| chr10:909683 \|910210 | hsa_circ_0017412 | LARP4B | 13 | 4 | 918.21 | 244.29 | -1.91 | Down | 0.01369 | 0.3939974 |
| chr7:18705836 \|18706099 | hsa_circ_0007904 | HDAC9 | 10 | 3 | 706.31 | 183.217 | -1.947 | Down | 0.02984 | 0.4821642 |
| chr10:126628943 \|126631876 | hsa_circ_0006545 | Y_RNA | 10 | 3 | 706.31 | 183.217 | -1.947 | Down | 0.02984 | 0.480266 |
| chr16:16218646 \|16219768 | hsa_circ_0000679 | ABCC1 | 10 | 3 | 706.31 | 183.217 | -1.947 | Down | 0.02984 | 0.4783826 |
| chr9:21476898 \|21477291 | hsa_circ_0008599 | MIR31HG | 10 | 3 | 706.31 | 183.217 | -1.947 | Down | 0.02984 | 0.4765139 |
| chr15:90760671 \|90763123 | hsa_circ_0036763 | SEMA4B | 17 | 5 | 1200.7 | 305.362 | -1.975 | Down | 0.00373 | 0.2586055 |
| chr9:87356807 \|87367000 | hsa_circ_0087378 | NTRK2 | 14 | 4 | 988.84 | 244.29 | -2.017 | Down | 0.00792 | 0.3268525 |
| chr2:36623757 \|36706837 | hsa_circ_0002348 | CRIM1 | 14 | 4 | 988.84 | 244.29 | -2.017 | Down | 0.00792 | 0.323584 |
| chr7:65705312 \|65713633 |  | snoU13, Y_RNA,  U3, SNORA51,  TPST1 | 8 | 2 | 565.05 | 122.145 | -2.21 | Down | 0.03721 | 0.5873556 |
| chr2:233599865 \|233626146 | hsa_circ_0007952 | GIGYF2 | 13 | 3 | 918.21 | 183.217 | -2.325 | Down | 0.00532 | 0.2898185 |
| chr1:12061458 \|12062160 | hsa_circ_0006470 | MFN2 | 13 | 3 | 918.21 | 183.217 | -2.325 | Down | 0.00532 | 0.2860051 |
| chr1:103444938 \|103455127 |  | COL11A1 | 13 | 3 | 918.21 | 183.217 | -2.325 | Down | 0.00532 | 0.2822907 |
| chr4:186168448 \|186188309 | hsa_circ_0004874 | SNX25 | 22 | 5 | 1553.9 | 305.362 | -2.347 | Down | 0.00022 | 0.027487 |
| chr2:242644068 \|242651486 | hsa_circ_0001124 | ING5 | 9 | 2 | 635.68 | 122.145 | -2.38 | Down | 0.02039 | 0.4210434 |
| chr1:208383625 \|208391347 | hsa_circ_0002472 | PLXNA2 | 15 | 3 | 1059.5 | 183.217 | -2.532 | Down | 0.0016 | 0.1358875 |
| chr16:14738131 \|14738466 | hsa_circ_0009065 | BFAR | 10 | 2 | 706.31 | 122.145 | -2.532 | Down | 0.01103 | 0.3667195 |
| chr12:117365828 \|117402659 | hsa_circ_0003677 | FBXW8 | 10 | 2 | 706.31 | 122.145 | -2.532 | Down | 0.01103 | 0.3637621 |
| chr8:74585342 \|74601048 | hsa_circ_0001811 | STAU2 | 11 | 2 | 776.95 | 122.145 | -2.669 | Down | 0.0059 | 0.3094742 |
| chr11:86778736 \|86802437 | hsa_circ_0008359 | TMEM135 | 11 | 2 | 776.95 | 122.145 | -2.669 | Down | 0.0059 | 0.3055568 |
| chr12:124071294 \|124074996 | hsa_circ_0000458 | TMED2 | 11 | 2 | 776.95 | 122.145 | -2.669 | Down | 0.0059 | 0.3017373 |
| chr1:41578955 \|41608784 | hsa_circ_0000063 | SCMH1 | 4 | 0 | 282.53 | 0.001 | -18.108 | Down | 0.04288 | 0.6590102 |
| chr1:92798948 \|92811471 | hsa_circ_0004463 | RPAP2 | 4 | 0 | 282.53 | 0.001 | -18.108 | Down | 0.04288 | 0.656542 |
| chr8:13356558 \|13357705 |  | DLC1 | 4 | 0 | 282.53 | 0.001 | -18.108 | Down | 0.04288 | 0.6540922 |
| chr2:26022254 \|26029206 | hsa_circ_0004476 | ASXL2 | 4 | 0 | 282.53 | 0.001 | -18.108 | Down | 0.04288 | 0.6516606 |
| chr3:47127685 \|47139571 |  | SETD2 | 4 | 0 | 282.53 | 0.001 | -18.108 | Down | 0.04288 | 0.6492471 |
| chr14:35438376 \|35441270 | hsa_circ_0008568 | RP11- 85K15.2 | 4 | 0 | 282.53 | 0.001 | -18.108 | Down | 0.04288 | 0.6468513 |
| chr3:37170554 \|37190529 | hsa_circ_0003264 | LRRFIP2 | 4 | 0 | 282.53 | 0.001 | -18.108 | Down | 0.04288 | 0.6444732 |
| chr15:43707792 \|43708614 | hsa_circ_0034982 | TP53BP1 | 4 | 0 | 282.53 | 0.001 | -18.108 | Down | 0.04288 | 0.6421125 |
| chr16:19775170 \|19775434 | hsa_circ_0004049 | IQCK | 4 | 0 | 282.53 | 0.001 | -18.108 | Down | 0.04288 | 0.639769 |
| chr5:78251118 \|78265015 | hsa_circ_0073119 | ARSB | 4 | 0 | 282.53 | 0.001 | -18.108 | Down | 0.04288 | 0.6374426 |
| chr6:126176166 \|126176386 |  | NCOA7 | 4 | 0 | 282.53 | 0.001 | -18.108 | Down | 0.04288 | 0.635133 |
| chr8:116599228 \|116599831 | hsa_circ_0085359 | TRPS1 | 4 | 0 | 282.53 | 0.001 | -18.108 | Down | 0.04288 | 0.6328401 |
| chr2:208841375 \|208842310 | hsa_circ_0001095 | PLEKHM3 | 4 | 0 | 282.53 | 0.001 | -18.108 | Down | 0.04288 | 0.6305637 |
| chr22:41349559 \|41360121 | hsa_circ_0008884 | RBX1, XPNPEP3 | 4 | 0 | 282.53 | 0.001 | -18.108 | Down | 0.04288 | 0.6283036 |
| chr17:40557254 \|40574872 |  | PTRF | 4 | 0 | 282.53 | 0.001 | -18.108 | Down | 0.04288 | 0.6260597 |
| chr11:9735013 \|9754257 |  | SWAP70 | 4 | 0 | 282.53 | 0.001 | -18.108 | Down | 0.04288 | 0.6238317 |
| chr1:197611841 \|197627499 | hsa_circ_0006324 | DENND1B | 4 | 0 | 282.53 | 0.001 | -18.108 | Down | 0.04288 | 0.6216195 |
| chr3:20017093 \|20017661 | hsa_circ_0064563 | RAB5A | 4 | 0 | 282.53 | 0.001 | -18.108 | Down | 0.04288 | 0.619423 |
| chr3:107429299 \|107435696 | hsa_circ_0001324 | BBX | 4 | 0 | 282.53 | 0.001 | -18.108 | Down | 0.04288 | 0.6172419 |
| chr7:1937836 \|1976533 | hsa_circ_0079136 | MAD1L1 | 4 | 0 | 282.53 | 0.001 | -18.108 | Down | 0.04288 | 0.6150762 |
| chr18:10534463 \|10550251 | hsa_circ_0004922 | NAPG | 4 | 0 | 282.53 | 0.001 | -18.108 | Down | 0.04288 | 0.6129256 |
| chrX:154736559 \|154754293 | hsa_circ_0007183 | TMLHE | 4 | 0 | 282.53 | 0.001 | -18.108 | Down | 0.04288 | 0.6107899 |
| chr22:29095826 \|29121355 |  | CHEK2 | 4 | 0 | 282.53 | 0.001 | -18.108 | Down | 0.04288 | 0.6086691 |
| chr5:178965995 \|178967833 |  | Y_RNA | 4 | 0 | 282.53 | 0.001 | -18.108 | Down | 0.04288 | 0.606563 |
| chr10:69773783 \|69797926 |  | HERC4 | 4 | 0 | 282.53 | 0.001 | -18.108 | Down | 0.04288 | 0.6044714 |
| chr3:49362135 \|49363278 | hsa_circ_0004609 | USP4 | 4 | 0 | 282.53 | 0.001 | -18.108 | Down | 0.04288 | 0.6023942 |
| chr14:67768106 \|67770316 | hsa_circ_0005429 | MPP5 | 4 | 0 | 282.53 | 0.001 | -18.108 | Down | 0.04288 | 0.6003312 |
| chr7:139746684 \|139757834 | hsa_circ_0082688 | PARP12 | 4 | 0 | 282.53 | 0.001 | -18.108 | Down | 0.04288 | 0.5982823 |
| chr1:152221371 \|152224234 |  | FLG-AS1 | 4 | 0 | 282.53 | 0.001 | -18.108 | Down | 0.04288 | 0.5962473 |
| chr16:1774558 \|1779579 | hsa_circ_0008480 | MAPK8IP3 | 4 | 0 | 282.53 | 0.001 | -18.108 | Down | 0.04288 | 0.5942261 |
| chr10:27453993 \|27454468 | hsa_circ_0000225 | MASTL | 4 | 0 | 282.53 | 0.001 | -18.108 | Down | 0.04288 | 0.5922186 |
| chr12:111990084 \|111990781 | hsa_circ_0028270 | ATXN2 | 4 | 0 | 282.53 | 0.001 | -18.108 | Down | 0.04288 | 0.5902246 |
| chr5:14673751 \|14681716 | hsa_circ_0005145 | FAM105B | 4 | 0 | 282.53 | 0.001 | -18.108 | Down | 0.04288 | 0.588244 |
| chr12:49880033 \|49884514 |  | SPATS2 | 4 | 0 | 282.53 | 0.001 | -18.108 | Down | 0.04288 | 0.5862766 |
| chr1:31810022 \|31811895 | hsa_circ_0009057 | ZCCHC17 | 4 | 0 | 282.53 | 0.001 | -18.108 | Down | 0.04288 | 0.5843224 |
| chr6:16326625 \|16328701 | hsa_circ_0007132 | ATXN1 | 4 | 0 | 282.53 | 0.001 | -18.108 | Down | 0.04288 | 0.5823811 |
| chr1:63269390 \|63286880 | hsa_circ_0012823 | ATG4C | 4 | 0 | 282.53 | 0.001 | -18.108 | Down | 0.04288 | 0.5804527 |
| chr17:131559 \|183725 | hsa_circ_0041151 | RPH3AL | 4 | 0 | 282.53 | 0.001 | -18.108 | Down | 0.04288 | 0.578537 |
| chr20:39704811 \|39713208 |  | TOP1 | 4 | 0 | 282.53 | 0.001 | -18.108 | Down | 0.04288 | 0.5766339 |
| chr8:135521862 \|135533244 | hsa_circ_0007934 | ZFAT | 4 | 0 | 282.53 | 0.001 | -18.108 | Down | 0.04288 | 0.5747433 |
| chr12:102107868 \|102110590 | hsa_circ_0006660 | CHPT1 | 4 | 0 | 282.53 | 0.001 | -18.108 | Down | 0.04288 | 0.5728651 |
| chr2:215609791 \|215634036 | hsa_circ_0007935 | BARD1 | 4 | 0 | 282.53 | 0.001 | -18.108 | Down | 0.04288 | 0.5709991 |
| chr1:741179 \|745550 | hsa_circ_0002333 | n/a | 4 | 0 | 282.53 | 0.001 | -18.108 | Down | 0.04288 | 0.5691452 |
| chr1:15964802 \|15978390 | hsa_circ_0000020 | DDI2 | 4 | 0 | 282.53 | 0.001 | -18.108 | Down | 0.04288 | 0.5673033 |
| chr12:112321385 \|112321572 | hsa_circ_0002488 | MAPKAPK5 | 4 | 0 | 282.53 | 0.001 | -18.108 | Down | 0.04288 | 0.5654733 |
| chr10:76598441 \|76603236 | hsa_circ_0005164 | KAT6B | 4 | 0 | 282.53 | 0.001 | -18.108 | Down | 0.04288 | 0.563655 |
| chr9:86292642 \|86301070 | hsa_circ_0003715 | UBQLN1 | 4 | 0 | 282.53 | 0.001 | -18.108 | Down | 0.04288 | 0.5618484 |
| chr19:38631824 \|38633350 | hsa_circ_0006670 | SIPA1L3 | 4 | 0 | 282.53 | 0.001 | -18.108 | Down | 0.04288 | 0.5600534 |
| chr3:183361268 \|183390272 | hsa_circ_0001368 | KLHL24 | 4 | 0 | 282.53 | 0.001 | -18.108 | Down | 0.04288 | 0.5582698 |
| chr6:57046830 \|57048709 |  | BAG2 | 4 | 0 | 282.53 | 0.001 | -18.108 | Down | 0.04288 | 0.5564975 |
| chr21:38792601 \|38798083 | hsa_circ_0004407 | snoU13, Y_RNA, DYRK1A | 4 | 0 | 282.53 | 0.001 | -18.108 | Down | 0.04288 | 0.5547364 |
| chr4:128995615 \|129012667 | hsa_circ_0070934 | LARP1B | 4 | 0 | 282.53 | 0.001 | -18.108 | Down | 0.04288 | 0.5529865 |
| chr5:108103794 \|108134090 |  | FER | 4 | 0 | 282.53 | 0.001 | -18.108 | Down | 0.04288 | 0.5512475 |
| chr3:52771602 \|52775515 | hsa_circ_0001309 | NEK4 | 4 | 0 | 282.53 | 0.001 | -18.108 | Down | 0.04288 | 0.5495195 |
| chr1:210003453 \|210004388 | hsa_circ_0016330 | DIEXF | 4 | 0 | 282.53 | 0.001 | -18.108 | Down | 0.04288 | 0.5478022 |
| chr1:21599192 \|21616649 | hsa_circ_0002402 | ECE1 | 4 | 0 | 282.53 | 0.001 | -18.108 | Down | 0.04288 | 0.5460957 |
| chr1:116202262 \|116206889 | hsa_circ_0002623 | VANGL1 | 4 | 0 | 282.53 | 0.001 | -18.108 | Down | 0.04288 | 0.5443997 |
| chr6:144858718 \|144872218 |  | UTRN | 4 | 0 | 282.53 | 0.001 | -18.108 | Down | 0.04288 | 0.5427143 |
| chr8:74585342 \|74652103 | hsa_circ_0084781 | STAU2 | 4 | 0 | 282.53 | 0.001 | -18.108 | Down | 0.04288 | 0.5410392 |
| chr18:42281140 \|42283072 | hsa_circ_0005942 | Y_RN,  snoU13, U3, SNORA73,  SETBP1 | 4 | 0 | 282.53 | 0.001 | -18.108 | Down | 0.04288 | 0.5393745 |
| chr4:39839476 \|39843676 |  | PDS5A | 4 | 0 | 282.53 | 0.001 | -18.108 | Down | 0.04288 | 0.53772 |
| chr20:50329505 \|50346517 | hsa_circ_0008694 | ATP9A | 4 | 0 | 282.53 | 0.001 | -18.108 | Down | 0.04288 | 0.5360756 |
| chr2:172193963 \|172196064 | hsa_circ_0057041 | METTL8 | 4 | 0 | 282.53 | 0.001 | -18.108 | Down | 0.04288 | 0.5344412 |
| chr12:459787 \|463400 | hsa_circ_0002881 | KDM5A | 4 | 0 | 282.53 | 0.001 | -18.108 | Down | 0.04288 | 0.5328167 |
| chr1:236332006 \|236341936 |  | GPR137B | 4 | 0 | 282.53 | 0.001 | -18.108 | Down | 0.04288 | 0.5312022 |
| chr12:82763166 \|82796909 |  | Y_RNA,  snoU13, U8, SNORA70, METTL25 | 4 | 0 | 282.53 | 0.001 | -18.108 | Down | 0.04288 | 0.5295973 |
| chr8:41832222 \|41845081 | hsa_circ_0084089 | KAT6A | 4 | 0 | 282.53 | 0.001 | -18.108 | Down | 0.04288 | 0.5280021 |
| chr18:59764914 \|59770135 |  | Y_RNA, snoU13, PIGN | 4 | 0 | 282.53 | 0.001 | -18.108 | Down | 0.04288 | 0.5264165 |
| chr1:35824526 \|35827390 | hsa_circ_0011536 | ZMYM4 | 4 | 0 | 282.53 | 0.001 | -18.108 | Down | 0.04288 | 0.5248405 |
| chr1:203676137 \|203677232 | hsa_circ_0007167 | ATP2B4 | 4 | 0 | 282.53 | 0.001 | -18.108 | Down | 0.04288 | 0.5232738 |
| chr1:14099573 \|14109326 |  | PRDM2 | 4 | 0 | 282.53 | 0.001 | -18.108 | Down | 0.04288 | 0.5217164 |
| chr12:42768665 \|42792796 | hsa_circ_0003961 | PPHLN1 | 4 | 0 | 282.53 | 0.001 | -18.108 | Down | 0.04288 | 0.5201683 |
| chr12:23887618 \|23908658 |  | SOX5 | 4 | 0 | 282.53 | 0.001 | -18.108 | Down | 0.04288 | 0.5186293 |
| chr19:19576149 \|19576423 |  | GATAD2A | 4 | 0 | 282.53 | 0.001 | -18.108 | Down | 0.04288 | 0.5170994 |
| chr7:22999875 \|23030758 | hsa_circ_0008951 | FAM126A | 4 | 0 | 282.53 | 0.001 | -18.108 | Down | 0.04288 | 0.5155786 |
| chr14:55168780 \|55204005 | hsa_circ_0002612 | SAMD4A | 4 | 0 | 282.53 | 0.001 | -18.108 | Down | 0.04288 | 0.5140666 |
| chr17:43195361 \|43198739 |  | PLCD3 | 4 | 0 | 282.53 | 0.001 | -18.108 | Down | 0.04288 | 0.5125635 |
| chr15:78337242 \|78346529 | hsa_circ_0036441 | TBC1D2B | 4 | 0 | 282.53 | 0.001 | -18.108 | Down | 0.04288 | 0.5110691 |
| chr17:60741909 \|60742310 | hsa_circ_0009052 | MRC2 | 4 | 0 | 282.53 | 0.001 | -18.108 | Down | 0.04288 | 0.5095835 |
| chr18:19345733 \|19371518 | hsa_circ_0004578 | MIB1 | 4 | 0 | 282.53 | 0.001 | -18.108 | Down | 0.04288 | 0.5081064 |
| chr3:122842183 \|122843190 |  | snoU13,  Y_RNA,  U3, SNORA25,  U8, PDIA5 | 4 | 0 | 282.53 | 0.001 | -18.108 | Down | 0.04288 | 0.5066379 |
| chr14:56119735 \|56125332 |  | KTN1 | 4 | 0 | 282.53 | 0.001 | -18.108 | Down | 0.04288 | 0.5051778 |
| chr11:128993341 \|129034322 | hsa_circ_0007843 | ARHGAP32 | 4 | 0 | 282.53 | 0.001 | -18.108 | Down | 0.04288 | 0.5037262 |
| chr16:23632683 \|23634451 | hsa_circ_0005582 | PALB2 | 4 | 0 | 282.53 | 0.001 | -18.108 | Down | 0.04288 | 0.5022828 |
| chr19:8533658 \|8539128 | hsa_circ_0003765 | HNRNPM | 4 | 0 | 282.53 | 0.001 | -18.108 | Down | 0.04288 | 0.5008477 |
| chr3:50677797 \|50679763 | hsa_circ_0065871 | MAPKAPK3 | 4 | 0 | 282.53 | 0.001 | -18.108 | Down | 0.04288 | 0.4994208 |
| chr20:45874752 \|45875261 | hsa_circ_0005996 | ZMYND8 | 4 | 0 | 282.53 | 0.001 | -18.108 | Down | 0.04288 | 0.498002 |
| chr14:96986392 \|96991728 | hsa_circ_0002120 | PAPOLA | 4 | 0 | 282.53 | 0.001 | -18.108 | Down | 0.04288 | 0.4965912 |
| chr21:17135210 \|17150346 | hsa_circ_0061278 | USP25 | 4 | 0 | 282.53 | 0.001 | -18.108 | Down | 0.04288 | 0.4951884 |
| chr1:108690901 \|108703915 | hsa_circ_0004270 | SLC25A24 | 4 | 0 | 282.53 | 0.001 | -18.108 | Down | 0.04288 | 0.4937936 |
| chr20:32207323 \|32211102 | hsa_circ_0003426 | CBFA2T2 | 4 | 0 | 282.53 | 0.001 | -18.108 | Down | 0.04288 | 0.4924065 |
| chr6:144858718 \|144898424 |  | UTRN | 4 | 0 | 282.53 | 0.001 | -18.108 | Down | 0.04288 | 0.4910272 |
| chr19:47865733 \|47865950 | hsa_circ_0051680 | DHX34 | 4 | 0 | 282.53 | 0.001 | -18.108 | Down | 0.04288 | 0.4896556 |
| chr16:30715385 \|30715636 | hsa_circ_0039076 | SRCAP | 4 | 0 | 282.53 | 0.001 | -18.108 | Down | 0.04288 | 0.4882917 |
| chr16:16205232 \|16205439 | hsa_circ_0038138 | ABCC1 | 4 | 0 | 282.53 | 0.001 | -18.108 | Down | 0.04288 | 0.4869353 |
| chr8:141710990 \|141716304 | hsa_circ_0005273 | PTK2 | 4 | 0 | 282.53 | 0.001 | -18.108 | Down | 0.04288 | 0.4855865 |
| chr15:85081763 \|85098301 |  | UBE2Q2P1 | 4 | 0 | 282.53 | 0.001 | -18.108 | Down | 0.04288 | 0.4842451 |
| chr17:131559 \|177370 | hsa_circ_0041150 | RPH3AL | 4 | 0 | 282.53 | 0.001 | -18.108 | Down | 0.04288 | 0.482911 |
| chr4:184104556 \|184114861 | hsa_circ_0003631 | Y_RNA,  snoU13, SNORA31,  WWC2 | 4 | 0 | 282.53 | 0.001 | -18.108 | Down | 0.04288 | 0.4815844 |
| chr7:98654828 \|98655174 | hsa_circ_0081323 | SMURF1 | 4 | 0 | 282.53 | 0.001 | -18.108 | Down | 0.04288 | 0.480265 |
| chr18:39617656 \|39629569 |  | PIK3C3 | 4 | 0 | 282.53 | 0.001 | -18.108 | Down | 0.04288 | 0.4789528 |
| chr3:185316200 \|185331196 |  | SENP2 | 4 | 0 | 282.53 | 0.001 | -18.108 | Down | 0.04288 | 0.4776477 |
| chr12:109210814 \|109217137 | hsa_circ_0028088 | SSH1 | 4 | 0 | 282.53 | 0.001 | -18.108 | Down | 0.04288 | 0.4763498 |
| chr10:31644076 \|31676727 | hsa_circ_0002765 | ZEB1 | 4 | 0 | 282.53 | 0.001 | -18.108 | Down | 0.04288 | 0.4750588 |
| chr3:48572945 \|48573896 | hsa_circ_0003091 | PFKFB4 | 4 | 0 | 282.53 | 0.001 | -18.108 | Down | 0.04288 | 0.4737749 |
| chr4:178274462 \|178281831 | hsa_circ_0001460 | NEIL3 | 4 | 0 | 282.53 | 0.001 | -18.108 | Down | 0.04288 | 0.4724979 |
| chr10:27821436 \|27822923 | hsa_circ_0000226 | RAB18 | 4 | 0 | 282.53 | 0.001 | -18.108 | Down | 0.04288 | 0.4712277 |
| chr19:20327949 \|20331226 | hsa_circ_0006455 | CTC-260 E6.6 | 4 | 0 | 282.53 | 0.001 | -18.108 | Down | 0.04288 | 0.4699644 |
| chr4:52729603 \|52765544 | hsa_circ_0069718 | DCUN1D4 | 4 | 0 | 282.53 | 0.001 | -18.108 | Down | 0.04288 | 0.4687078 |
| chr15:63824846 \|63855207 | hsa_circ_0002138 | USP3 | 4 | 0 | 282.53 | 0.001 | -18.108 | Down | 0.04288 | 0.4674579 |
| chr20:13251151 \|13269330 |  | ISM1 | 4 | 0 | 282.53 | 0.001 | -18.108 | Down | 0.04288 | 0.4662147 |
| chr3:47676680 \|47677626 | hsa_circ_0065251 | SMARCC1 | 5 | 0 | 353.16 | 0.001 | -18.43 | Down | 0.01988 | 0.5383241 |
| chr9:79244108 \|79259828 |  | PRUNE2 | 5 | 0 | 353.16 | 0.001 | -18.43 | Down | 0.01988 | 0.5347825 |
| chr20:17937577 \|17941969 | hsa_circ_0009173 | SNX5 | 5 | 0 | 353.16 | 0.001 | -18.43 | Down | 0.01988 | 0.5312872 |
| chr3:136323151 \|136323315 | hsa_circ_0001342 | STAG1 | 5 | 0 | 353.16 | 0.001 | -18.43 | Down | 0.01988 | 0.5278373 |
| chr17:59853762 \|59857762 | hsa_circ_0006968 | BRIP1 | 5 | 0 | 353.16 | 0.001 | -18.43 | Down | 0.01988 | 0.5244318 |
| chr4:170458960 \|170477246 | hsa_circ_0003196 | NEK1 | 5 | 0 | 353.16 | 0.001 | -18.43 | Down | 0.01988 | 0.5210701 |
| chr5:80908381 \|80946158 |  | Y_RNA,  RPS23P5, snoU13, SNORA40, U3, SNORA57,  SNORA31,  SSBP2 | 5 | 0 | 353.16 | 0.001 | -18.43 | Down | 0.01988 | 0.5177512 |
| chr3:128984392 \|128985942 | hsa_circ_0004271 | COPG1 | 5 | 0 | 353.16 | 0.001 | -18.43 | Down | 0.01988 | 0.5144743 |
| chr12:70193989 \|70195501 | hsa_circ_0000419 | RAB3IP | 5 | 0 | 353.16 | 0.001 | -18.43 | Down | 0.01988 | 0.5112386 |
| chr14:97299804 \|97327072 | hsa_circ_0000566 | VRK1 | 5 | 0 | 353.16 | 0.001 | -18.43 | Down | 0.01988 | 0.5080434 |
| chr9:136277419 \|136278041 | hsa_circ_0089371 | REXO4 | 5 | 0 | 353.16 | 0.001 | -18.43 | Down | 0.01988 | 0.5048878 |
| chr4:122591109 \|122602916 | hsa_circ_0070822 | ANXA5 | 5 | 0 | 353.16 | 0.001 | -18.43 | Down | 0.01988 | 0.5017712 |
| chr19:45781181 \|45783992 | hsa_circ_0004440 | MARK4 | 5 | 0 | 353.16 | 0.001 | -18.43 | Down | 0.01988 | 0.4986929 |
| chr21:33750754 \|33756773 | hsa_circ_0001183 | URB1 | 5 | 0 | 353.16 | 0.001 | -18.43 | Down | 0.01988 | 0.4956521 |
| chr3:11399892 \|11468400 | hsa_circ_0008210 | ATG7 | 5 | 0 | 353.16 | 0.001 | -18.43 | Down | 0.01988 | 0.4926481 |
| chr7:158590633 \|158591763 | hsa_circ_0083225 | ESYT2 | 5 | 0 | 353.16 | 0.001 | -18.43 | Down | 0.01988 | 0.4896803 |
| chr15:41961026 \|41962156 | hsa_circ_0000591 | MGA | 5 | 0 | 353.16 | 0.001 | -18.43 | Down | 0.01988 | 0.4867481 |
| chr4:38091553 \|38104778 | hsa_circ_0001402 | TBC1D1 | 5 | 0 | 353.16 | 0.001 | -18.43 | Down | 0.01988 | 0.4838508 |
| chr7:24659671 \|24690331 | hsa_circ_0004582 | MPP6 | 5 | 0 | 353.16 | 0.001 | -18.43 | Down | 0.01988 | 0.4809878 |
| chr2:36605500 \|36669878 | hsa_circ_0007972 | CRIM1 | 5 | 0 | 353.16 | 0.001 | -18.43 | Down | 0.01988 | 0.4781585 |
| chr11:63960550 \|63965446 | hsa_circ_0022631 | STIP1 | 5 | 0 | 353.16 | 0.001 | -18.43 | Down | 0.01988 | 0.4753622 |
| chr3:185638892 \|185639914 | hsa_circ_0006840 | TRA2B | 5 | 0 | 353.16 | 0.001 | -18.43 | Down | 0.01988 | 0.4725985 |
| chr3:196533450 \|196539722 | hsa_circ_0004950 | PAK2 | 5 | 0 | 353.16 | 0.001 | -18.43 | Down | 0.01988 | 0.4698667 |
| chr2:110321943 \|110350696 | hsa_circ_0002076 | SEPT10 | 5 | 0 | 353.16 | 0.001 | -18.43 | Down | 0.01988 | 0.4671663 |
| chr18:46855943 \|46860223 |  | DYM | 5 | 0 | 353.16 | 0.001 | -18.43 | Down | 0.01988 | 0.4644968 |
| chr11:19901415 \|19914140 | hsa_circ_0006412 | NAV2 | 5 | 0 | 353.16 | 0.001 | -18.43 | Down | 0.01988 | 0.4618576 |
| chr18:47017996 \|47018203 | hsa_circ_0007956 | RPL17 | 5 | 0 | 353.16 | 0.001 | -18.43 | Down | 0.01988 | 0.4592482 |
| chr17:34864901 \|34867294 | hsa_circ_0003507 | MYO19 | 5 | 0 | 353.16 | 0.001 | -18.43 | Down | 0.01988 | 0.4566682 |
| chr10:75842212 \|75868914 |  | VCL | 5 | 0 | 353.16 | 0.001 | -18.43 | Down | 0.01988 | 0.454117 |
| chr2:26505713 \|26505919 |  | HADHB | 5 | 0 | 353.16 | 0.001 | -18.43 | Down | 0.01988 | 0.4515941 |
| chr6:170852689 \|170858201 | hsa_circ_0006107 | PSMB1 | 5 | 0 | 353.16 | 0.001 | -18.43 | Down | 0.01988 | 0.4490991 |
| chr13:52971367 \|52976816 | hsa_circ_0008581 | THSD1 | 5 | 0 | 353.16 | 0.001 | -18.43 | Down | 0.01988 | 0.4466315 |
| chr3:56661065 \|56662642 | hsa_circ_0001314 | FAM208A | 5 | 0 | 353.16 | 0.001 | -18.43 | Down | 0.01988 | 0.4441909 |
| chr13:76195899 \|76335174 |  | RP11-29 G8.3 | 5 | 0 | 353.16 | 0.001 | -18.43 | Down | 0.01988 | 0.4417768 |
| chr5:31448654 \|31451747 | hsa_circ_0004599 | DROSHA | 5 | 0 | 353.16 | 0.001 | -18.43 | Down | 0.01988 | 0.4393888 |
| chr9:86297866 \|86301070 | hsa_circ_0005142 | UBQLN1 | 5 | 0 | 353.16 | 0.001 | -18.43 | Down | 0.01988 | 0.4370265 |
| chr7:38829401 \|38836464 | hsa_circ_0002808 | VPS41 | 5 | 0 | 353.16 | 0.001 | -18.43 | Down | 0.01988 | 0.4346895 |
| chr10:135209217 \|135216277 | hsa_circ_0000271 | RP11-108 K14.8, MTG1 | 5 | 0 | 353.16 | 0.001 | -18.43 | Down | 0.01988 | 0.4323773 |
| chr2:9458653 \|9468040 | hsa_circ_0002362 | ASAP2 | 5 | 0 | 353.16 | 0.001 | -18.43 | Down | 0.01988 | 0.4300896 |
| chr8:62531537 \|62566219 | hsa_circ_0084606 | ASPH | 5 | 0 | 353.16 | 0.001 | -18.43 | Down | 0.01988 | 0.427826 |
| chr22:41670604 \|41677086 | hsa_circ_0001235 | RANGAP1 | 5 | 0 | 353.16 | 0.001 | -18.43 | Down | 0.01988 | 0.4255861 |
| chr5:102432245 \|102433485 | hsa_circ_0006499 | GIN1 | 5 | 0 | 353.16 | 0.001 | -18.43 | Down | 0.01988 | 0.4233695 |
| chr9:134518626 \|134526336 | hsa_circ_0089254 | RAPGEF1 | 5 | 0 | 353.16 | 0.001 | -18.43 | Down | 0.01988 | 0.4211758 |
| chr10:88218746 \|88233730 |  | WAPAL | 5 | 0 | 353.16 | 0.001 | -18.43 | Down | 0.01988 | 0.4190048 |
| chr2:9083316 \|9098771 | hsa_circ_0007334 | MBOAT2 | 5 | 0 | 353.16 | 0.001 | -18.43 | Down | 0.01988 | 0.4168561 |
| chr2:36596251 \|36623930 | hsa_circ_0004182 | snoU13,  Y_RNA, SNORA51,  SNORA40,  SNORD112,  CRIM1 | 5 | 0 | 353.16 | 0.001 | -18.43 | Down | 0.01988 | 0.4147293 |
| chr7:80447615 \|80458061 | hsa_circ_0080910 | SEMA3C | 5 | 0 | 353.16 | 0.001 | -18.43 | Down | 0.01988 | 0.412624 |
| chr1:27267948 \|27268309 | hsa_circ_0003940 | NUDC | 6 | 0 | 423.79 | 0.001 | -18.693 | Down | 0.00922 | 0.3695448 |
| chr1:117944808 \|118009049 | hsa_circ_0000120 | MAN1A2 | 6 | 0 | 423.79 | 0.001 | -18.693 | Down | 0.00922 | 0.365957 |
| chr1:25666965 \|25669564 | hsa_circ_0000032 | TMEM50A | 6 | 0 | 423.79 | 0.001 | -18.693 | Down | 0.00922 | 0.3624381 |
| chr3:141811903 \|141820683 | hsa_circ_0008304 | TFDP2 | 6 | 0 | 423.79 | 0.001 | -18.693 | Down | 0.00922 | 0.3589864 |
| chr11:581492 \|592674 |  | PHRF1 | 6 | 0 | 423.79 | 0.001 | -18.693 | Down | 0.00922 | 0.3555997 |
| chr10:69726440 \|69773943 |  | HERC4 | 6 | 0 | 423.79 | 0.001 | -18.693 | Down | 0.00922 | 0.3522763 |
| chr5:56542127 \|56546968 | hsa_circ_0072547 | GPBP1 | 6 | 0 | 423.79 | 0.001 | -18.693 | Down | 0.00922 | 0.3490145 |
| chrY:13688616 \|13851691 |  | n/a | 6 | 0 | 423.79 | 0.001 | -18.693 | Down | 0.00922 | 0.3458125 |
| chr2:109388157 \|109389502 | hsa_circ_0006965 | RANBP2 | 6 | 0 | 423.79 | 0.001 | -18.693 | Down | 0.00922 | 0.3426688 |
| chr18:9208655 \|9221997 | hsa_circ_0003652 | ANKRD12 | 6 | 0 | 423.79 | 0.001 | -18.693 | Down | 0.00922 | 0.3395817 |
| chr5:36953720 \|36976504 | hsa_circ_0001472 | NIPBL | 6 | 0 | 423.79 | 0.001 | -18.693 | Down | 0.00922 | 0.3365497 |
| chr12:96717726 \|96728643 | hsa_circ_0002762 | CDK17 | 6 | 0 | 423.79 | 0.001 | -18.693 | Down | 0.00922 | 0.3335714 |
| chr12:122825300 \|122826244 | hsa_circ_0029069 | CLIP1 | 6 | 0 | 423.79 | 0.001 | -18.693 | Down | 0.00922 | 0.3306453 |
| chr10:126727566 \|126799662 | hsa_circ_0005418 | CTBP2 | 6 | 0 | 423.79 | 0.001 | -18.693 | Down | 0.00922 | 0.3277702 |
| chr1:243579004 \|243589860 | hsa_circ_0017241 | SDCCAG8 | 6 | 0 | 423.79 | 0.001 | -18.693 | Down | 0.00922 | 0.3249445 |
| chr2:63206323 \|63223901 | hsa_circ_0005552 | EHBP1 | 6 | 0 | 423.79 | 0.001 | -18.693 | Down | 0.00922 | 0.3221672 |
| chr4:148785997 \|148803083 | hsa_circ_0008243 | ARHGAP10 | 6 | 0 | 423.79 | 0.001 | -18.693 | Down | 0.00922 | 0.319437 |
| chr15:99250791 \|99251336 | hsa_circ_0005035 | IGF1R | 6 | 0 | 423.79 | 0.001 | -18.693 | Down | 0.00922 | 0.3167527 |
| chr7:102039995 \|102047945 | hsa_circ_0007004 | PRKRIP1 | 6 | 0 | 423.79 | 0.001 | -18.693 | Down | 0.00922 | 0.3141131 |
| chr16:1675974 \|1682366 | hsa_circ_0005606 | CRAMP1L | 6 | 0 | 423.79 | 0.001 | -18.693 | Down | 0.00922 | 0.3115171 |
| chr21:47409034 \|47410336 |  | COL6A1 | 7 | 0 | 494.42 | 0.001 | -18.915 | Down | 0.00428 | 0.2648318 |
| chr2:135010666 \|135028121 | hsa_circ_0002480 | MGAT5 | 7 | 0 | 494.42 | 0.001 | -18.915 | Down | 0.00428 | 0.2608791 |
| chr7:34006091 \|34014396 | hsa_circ_0004750 | BMPER | 7 | 0 | 494.42 | 0.001 | -18.915 | Down | 0.00428 | 0.2570426 |
| chr11:77336008 \|77336863 | hsa_circ_0000343 | CLNS1A | 7 | 0 | 494.42 | 0.001 | -18.915 | Down | 0.00428 | 0.2533174 |
| chr3:27363668 \|27398395 | hsa_circ_0005018 | snoU13,  Y_RNA,  NEK10 | 7 | 0 | 494.42 | 0.001 | -18.915 | Down | 0.00428 | 0.2496985 |
| chr3:63884075 \|63938159 | hsa_circ_0002634 | ATXN7 | 7 | 0 | 494.42 | 0.001 | -18.915 | Down | 0.00428 | 0.2461817 |
| chr1:235993526 \|235996967 | hsa_circ_0005899 | LYST | 7 | 0 | 494.42 | 0.001 | -18.915 | Down | 0.00428 | 0.2427625 |
| chr18:77170403 \|77171501 | hsa_circ_0048023 | NFATC1 | 7 | 0 | 494.42 | 0.001 | -18.915 | Down | 0.00428 | 0.239437 |
| chr4:83799883 \|83803093 | hsa_circ_0006618 | SEC31A | 8 | 0 | 565.05 | 0.001 | -19.108 | Down | 0.00198 | 0.1654112 |
| chr10:69773783 \|69804320 | hsa_circ_0003259 | HERC4 | 8 | 0 | 565.05 | 0.001 | -19.108 | Down | 0.00198 | 0.1621029 |
| chr11:110007388 \|110030215 | hsa_circ_0003541 | ZC3H12C | 8 | 0 | 565.05 | 0.001 | -19.108 | Down | 0.00198 | 0.1589244 |
| chr14:74973401 \|74974013 |  | LTBP2 | 8 | 0 | 565.05 | 0.001 | -19.108 | Down | 0.00198 | 0.1558682 |
| chr8:18622959 \|18662408 | hsa_circ_0002111 | PSD3 | 8 | 0 | 565.05 | 0.001 | -19.108 | Down | 0.00198 | 0.1529273 |
| chr10:15875629 \|15889942 | hsa_circ_0006665 | FAM188A | 8 | 0 | 565.05 | 0.001 | -19.108 | Down | 0.00198 | 0.1500953 |
| chr10:123298106 \|123325218 |  | FGFR2 | 8 | 0 | 565.05 | 0.001 | -19.108 | Down | 0.00198 | 0.1473663 |
| chrX:37245852 \|37285253 | hsa_circ_0008077 | PRRG1 | 9 | 0 | 635.68 | 0.001 | -19.278 | Down | 0.00092 | 0.0874056 |
| chr11:9225207 \|9229179 | hsa_circ_0004099 | DENND5A | 9 | 0 | 635.68 | 0.001 | -19.278 | Down | 0.00092 | 0.0854191 |
| chr1:146757975 \|146759412 | hsa_circ_0006296 | CHD1L | 9 | 0 | 635.68 | 0.001 | -19.278 | Down | 0.00092 | 0.0835209 |
| chr16:31102096 \|31102663 | hsa_circ_0006719 | Y_RNA,  SNORA48, snoU13, RP11-196G11.1 | 10 | 0 | 706.31 | 0.001 | -19.43 | Down | 0.00043 | 0.0471035 |
| chr8:42914235 \|42919358 | hsa_circ_0001796 | RP11-598 P20.5, FNTA | 12 | 0 | 847.58 | 0.001 | -19.693 | Down | 9.17E-05 | 0.0124918 |
| chr15:93540187 \|93541851 | hsa_circ_0000655 | CHD2 | 12 | 0 | 847.58 | 0.001 | -19.693 | Down | 9.17E-05 | 0.0120889 |
| chr1:16891302 \|16893846 |  | NBPF1 | 13 | 0 | 918.21 | 0.001 | -19.808 | Down | 4.25E-05 | 0.0062063 |
| chr1:32381496 \|32385259 | hsa_circ_0007364 | PTP4A2 | 13 | 0 | 918.21 | 0.001 | -19.808 | Down | 4.25E-05 | 0.0059923 |
| chr16:80718435 \|80719026 | hsa_circ_0004087 | CDYL2 | 15 | 0 | 1059.5 | 0.001 | -20.015 | Down | 9.14E-06 | 0.0014947 |
| chr4:54249940 \|54256040 | hsa_circ_0001411 | FIP1L1 | 18 | 0 | 1271.4 | 0.001 | -20.278 | Down | 9.11E-07 | 0.0001774 |
| chr22:42575615 \|42611347 |  | TCF20 | 18 | 0 | 1271.4 | 0.001 | -20.278 | Down | 9.11E-07 | 0.0001694 |
| chr3:27490142 \|27493989 | hsa_circ_0001277 | SLC4A7 | 21 | 0 | 1483.3 | 0.001 | -20.5 | Down | 9.09E-08 | 1.96E-05 |
| chr6:42559889 \|42562042 | hsa_circ_0001603 | UBR2 | 22 | 0 | 1553.9 | 0.001 | -20.567 | Down | 4.21E-08 | 1.08E-05 |
| chr19:41754419 \|41754725 | hsa_circ_0002882 | AXL | 25 | 0 | 1765.8 | 0.001 | -20.752 | Down | 4.20E-09 | 1.23E-06 |
| chr9:99220661 \|99220823 | hsa_circ_0009125 | HABP4 | 27 | 0 | 1907 | 0.001 | -20.863 | Down | 9.04E-10 | 2.84E-07 |
| chr21:35475134 \|35497780 | hsa_circ_0005076 | MRPS6 | 28 | 0 | 1977.7 | 0.001 | -20.915 | Down | 4.19E-10 | 1.43E-07 |
| chr11:12785726 \|12785981 |  | TEAD1 | 32 | 0 | 2260.2 | 0.001 | -21.108 | Down | 1.94E-11 | 8.80E-09 |
| chr1:27269151 \|27269556 | hsa_circ_0005087 | NUDC | 33 | 0 | 2330.8 | 0.001 | -21.152 | Down | 8.98E-12 | 4.59E-09 |
| chr14:55168780 \|55169298 | hsa_circ_0004846 | SAMD4A | 34 | 0 | 2401.5 | 0.001 | -21.195 | Down | 4.17E-12 | 2.43E-09 |
| chr20:30954187 \|30959677 | hsa_circ_0006922 | ASXL1 | 42 | 0 | 2966.5 | 0.001 | -21.5 | Down | 8.91E-15 | 6.07E-12 |
| chr3:196118684 \|196129890 | hsa_circ_0001380 | UBXN7 | 47 | 0 | 3319.7 | 0.001 | -21.663 | Down | 1.91E-16 | 1.56E-13 |
| chr1:155582209 \|155717687 |  | MSTO1 | 52 | 0 | 3672.8 | 0.001 | -21.808 | Down | 4.09E-18 | 4.18E-15 |
| chr3:145838899 \|145842016 |  | PLOD2 | 66 | 0 | 4661.7 | 0.001 | -22.152 | Down | 8.70E-23 | 1.78E-19 |

NC: negative control of osteoarthritis; OA: osteoarthritis.
